# Synthesis and biological evaluation of new nanosized aromatic polyamides containing amido- and sulfonamidopyrimidines pendant structures

**DOI:** 10.1186/s13065-015-0123-2

**Published:** 2015-08-19

**Authors:** Hammed H A M Hassan, Elsayed M E Mansour, Asmaa M S Abou Zeid, Ehab R El-Helow, Amel F Elhusseiny, Raafat Soliman

**Affiliations:** Department of Chemistry, Faculty of Science, Alexandria University, Ibrahimia, P. O. Box 426, Alexandria, 21321 Egypt; Department of Microbiology and Immunology, Faculty of Pharmacy, Pharos University, Canal El Mahmoudia Street, Alexandria, 21311 Egypt; Department of Pharmaceutical Chemistry, Faculty of Pharmacy, Alexandria University, Alexandria, Egypt

**Keywords:** Synthesis, Polymers, Sulfonamides, Pyrimidines, Microbial activity

## Abstract

**Background:**

Antibiotics are biocides or products that inhibit the growth of microorganisms in the living cells and there are extensive works directed to develop efficient antimicrobial agents. The sulfonamide-containing polymers have great potential to resist gram-positive or gram-negative bacterial and fungal attacks. As a therapeutic agent, the sulfonamides have been reported as antitumor and antimicrobial agents against bacteria, being more potent against gram positive rather than gram negative strains. Design of new classes of inhibitors bearing fluorescent tails, as therapeutic and imaging agents, is currently an active area of research. Here, we describe the synthesis of a new family of polyamides based on chlorophenyl-3,5-diaminobenzamides, methyl substituted pyrimidinoamido-3,5-diamino- benzamides and methyl substituted pyrimidinosulfonamido-3,5-diaminobenzamides and evaluation of their thermal, optical and antimicrobial properties.

**Results:**

We report the synthesis of a new series of nanosized polyamides containing bioactive pendent structures. The spherical nanosized polymer particles are soluble in many organic solvents and exhibited emissions ranging from blue to orange wavelength depending on the nature of the signaling unit. Pyrimidine- and *p*-chloroaromatic containing polymers exhibited higher bioactivity than that contain the sulfonamide group. The amidopyrimidine polymers exhibited remarkable antifungal and antibacterial activity and thus, these types of polymers are promising candidates for biomedical applications.

**Conclusions:**

The SEM analysis indicated that most of the polyamides were organized as well defined nano sized spheres, but in certain derivatives small amount of aggregated nanospheres were also observed. Thermal analyses were studied up to 700 °C and results showed comparable thermal behavior. The optical results revealed that polymeric series (A) exhibited orange emission, series (B) showed green emission while series (C) exhibited yellow and blue emissions. Benzene/pyridine structure interchange resulted in red shifted peaks attributed to the localized lone pair of electrons on a nitrogen atom which offer a greater electron affinity and better electron-transporting properties. The amido- and sulfonamide pyrimidine containing polymers exhibited the most potent antimicrobial activity. Relative to the reference *Gentamicin*, the polymer **54** exhibited comparable antibacterial activity against gram negative bacteria. Analogues **52** and **57** exhibited remarkable antibacterial activities compared to the references used. Thus, these polyamides are likely to be promising broad spectrum antibacterial agents and deserve further investigation at the molecular level.

**Graphical abstract:**
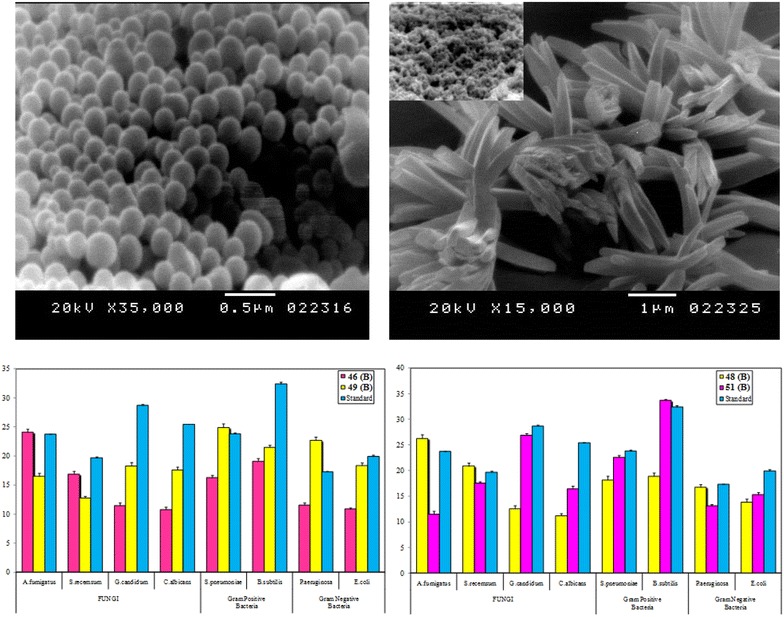
The synthesis and characterization of a new series of nanosized polyamides containing chloroaromatic (A), pyrimidinoamido- (B) and pyrimidosulfonamido- (C) pendent structures as promising candidates for biomedical applications is described.

**Electronic supplementary material:**

The online version of this article (doi:10.1186/s13065-015-0123-2) contains supplementary material, which is available to authorized users.

## Background

Polymer–drug structures are currently known constructs that chemically combine the bioactive part with a specific region of the polymer to ensure its delivery to the targeted intracellular compartment [1]. Several synthetic approaches have been published to bond the polymer and drug either in the polymer backbone or in the side-chain [2–4]. The development of longer term bioactive antimicrobial polymers is a research area focused on solving microorganism’s contamination problems [5]. Polymer modifications to achieve such activity include incorporation of known antimicrobial compounds such as hydantoins, glycolylureas, imidazolidinones and oxazolidinones [6–8]. In addition, several studies have reported the incorporation of a pyrimidine ring into the polymeric backbone. Incorporation of pyrimidine nuclei modify the polymer’s solubility and processability due to the possibility of protonation and/or alkylation of the lone pair. Moreover, the electronegative nitrogen atoms offers many substituted pyrimidine structures through direct substitution reactions [9–12].

Sulfonamides have been used in therapeutics for many years [13, 14]. The sulfonamide derivatives have been reported to show substantial antitumor activity in vitro and/or in vivo [15–18], HIV protease inhibitors [19, 20] and cell entry [21]. The polysulfonamide is an active agent that shields the toxic polycations. The copolymers possess higher activity toward fungi than against bacteria and being more gram positive rather than gram negative as it is common. A novel strategy for cancer treatment based on a new class of inhibitors bearing fluorescent tails is currently an active research area for use therapeutic and imaging agents for poorly responsive tumors to classical chemo- and radiotherapies. For instance, a bioactive novel fluorescent fluoro poly(amide–sulfonamide)s possessed distinctive structure as well as unique properties were reported [22].

We previously succeeded in preparing nanosized aromatic polyamides with remarkable electrical and biomedical properties [23–29]. Herein, we describe the preparation of novel nanosized aromatic polyamides with bioactive pendant structures comprised of substituted pyrimidines that act as signaling units due to their fluorescent and chromogenic characteristics. We report the synthesis of novel diamine types derived from isomeric chlorophenyl-3,5-dinitrobenzamides, isomeric methyl substituted pyrimidinoamido-3,5-dinitrobenzamides and isomeric methyl substituted pyrimidinosulfonamido-3,5-dinitrobenzamides. Subsequent reactions of these diamines with the readily available isophthaloyl chloride or pyridine-2,6-dicarbonyl dichloride furnished a new series of bioactive, fluorescent aromatic polyamides containing chloroaromatic, pyrimidinoamido-, pyrimidine- sulfonamido pendent structures, respectively. Evaluation of thermal, optical and antimicrobial properties of the prepared polymers are also described.

## Experimental

### General

Melting points were determined with an electrothermal melting point apparatus and are not corrected. Infrared spectra (IR, KBr pellets; 3 mm thickness) were recorded on a Perkin-Elmer Infrared Spectrophotometer (FT-IR 1650). All spectra were recorded within the wave number range of 600–4,000 cm^−1^ at 25 °C. ^1^H-NMR and ^13^C-NMR spectra were recorded using the JEOL 500 mHZ spectrometer operating in DMSO-d_6_ and expressed on the δ scale ppm. Absorption spectra were measured with a UV 500 UV–vis spectrometer at room temperature (r.t) in DMSO with a polymer concentration of 2 mg/10 ml. Inherent viscosities (*η*_inh_) were measured at a concentration of 0.5 g/100 dL in DMSO at 30 °C by using an Ubbelohde Viscometer. Differential thermo gravimetric (DTG) analyses were carried out in the temperature range from 20 to 700 °C in a steam of nitrogen atmosphere by a Shimadzu DTG 60H thermal analyzer. The experimental conditions were: platinum crucible, nitrogen atmosphere with a 30 ml/min flow rate and a heating rate 20 °C/min. Thermo gravimetric (TGA) analyses were carried out using SDTQ600-V20.5-Build-15. (DTG), (TGA) and elemental analyses were performed at the Microanalytical Unit, Cairo University. The morphologies of polymer nanoparticles were observed by Scanning Electron Microscope (SEM) (JEOL-JSM5300), at the E-Microscope Unit; Faculty of Science, Alexandria University. The samples were sonicated in de-ionized water for 5 min and deposited onto carbon coated copper mesh and allowed to air-dry before the examination. The antimicrobial activities were carried out using diffusion agar techniques and the evaluation of cytotoxicity against HepG-2, HCT-116 and MCF-7 cell lines were performed at the antimicrobial unit and the regional center of mycology and biotechnology, Al-Azhar University, Cairo.

### Synthesis of 3,5-diaminobenzamide containing pendent aromatic structures **8**–**12**, **16**–**18** and **22**–**24** (General method)

The appropriate commercial amine **3**–**7**, **18**–**20** and **27**–**29** (13.05 mmol) dissolved in DMF (20 ml) was treated with 3,5 dinitrobenzoyl chloride **2** (13.05 mmol). The mixture was stirred for 20 h at r.t and then it was poured into cold water and filtered and the obtained 3,5-dinitrobenzamides were dried in a vacuum oven at 60 °C. The following data were recorded: *3,5*-*Dinitro*-*N*-*phenylbenzamide***8**: Yield: (3 g, 80 %), m.p. 226 °C. IR (ν, cm^−1^): 3,461, 3,290 (NH_str_), 3,104 (CH_str_ arom), 1,653 (C=O_str_ amide), 1,600 (C=C_str_ arom), 1,537 (NO_2asym str_), 1,494, 1,441, 1,340 (NO_2sym str_), 1,272, 1,162, 1,108, 1,076, 948, 916, 862, 816, 760, 726, 692, 568, 512. ^1^H-NMR (500 MHz, DMSO): δ 9.14 (s, 1H, CONH), 9.11 (s, 2H, H5, H6 arom), 8.97 (s, 1H, H2 arom.), 7.76–7.13 (m, 5H, C6H5). Elemental analysis calculated for C_13_H_9_N_3_O_5_: C, 54.35; H, 3.13; N, 14.63. Found: C, 54.71; H, 3.50; N, 14.91.

*N*-*(2*-*Chlorophenyl)*-*3,5*-*dinitrobenzamide***9**: Yield: (6.1 g, 73 %), m.p. 204 °C. IR (ν, cm^−1^): 3,448, 3,253 (NH_str_), 3,099 (CH_str_ arom), 1,657 (C=O_str_ amide), 1,589 (C=C_str_ arom), 1,537 (NO_2asym str_), 1,471, 1,436, 1,343 (NO_2sym str_), 1,307, 1,270, 1,163, 1,106, 1,077, 917, 823, 765 (C–Cl_str_), 748, 676, 572. Elemental analysis calculated for C_13_H_8_ClN_3_O_5_: C, 48.50; H, 2.48; N, 13.06. Found: C, 48.21; H, 2.63; N, 13.29.

*N*-*(3*-*Chlorophenyl)*-*3,5*-*dinitrobenzamide***10**: Yield: (6.8 g, 81.2 %), m.p. 219 °C. IR (ν, cm^−1^): 3,429, 3,281 (N–H_str_), 3,100 (C–H_str_ aromatic), 1,663 (C=O_str_ amide), 1,628, 1,595 (C=C_str_ arom), 1,541 (NO_2asym str_), 1,477, 1,421, 1,344 (NO_2sym str_), 1,304, 1,257, 1,163, 1,079, 1,005, 917, 883 (C–Cl_str_), 833, 790, 726, 689, 567, 534. Elemental analysis calculated for C_13_H_8_ClN_3_O_5_: C, 48.50; H, 2.48; N, 13.06. Found: C, 48.73; H, 2.66; N, 13.32.

*N*-*(4*-*Chlorophenyl)*-*3,5*-*dinitrobenzamide***11**: Yield: (2.6 g, 62 %), m.p. 198 °C. IR (ν, cm^−1^): 3,419, 3,270 (NH_str_), 3,182, 3,097 (C–H_str_ arom), 2,920, 1,653 (C=O_str_ amide), 1,598 (C=C_str_ arom), 1,543 (NO_2__asym str_), 1,493, 1,397, 1,343 (NO_2symstr_), 1,263, 1,163, 1,164, 1,090, 1,013, 955, 918, 869 (C–Cl_str_), 827, 707, 509. Elemental analysis calculated for C_13_H_8_ClN_3_O_5_: C, 48.50; H, 2.48; N, 13.06. Found: C, 48.22; H, 2.18; N, 13.36.

*N*-*(4*-*(N*-*(2*-*Chlorophenyl)sulfamoyl) phenyl)*-*3,5*-*dinitrobenzamide***12**: Yield: (3.6 g, 57 %), m.p. 210 °C. IR (ν, cm^−1^): 3,361 (NH_str_ SO_2_NH), 3,254 (NH_str_ CONH), 3,090 (C–H_str_ arom), 1,691, 1,628 (C=O_str_ amide), 1,593 (C=C_str_ arom), 1,537 (NO_2 str_), 1,482, 1,398, 1,339 (SO_2 asymstr_), 1,264, 1,158 (SO_2symstr_), 1,087, 912, 835, 759 (C-Cl_str_), 724, 684, 638, 565, 449. Elemental analysis calculated for C_19_H_13_ClN_4_O_7_S: C, 47.84; H, 2.73; N, 11.75; S, 6.71. Found: C, 47.37; H, 2.99; N, 11.47; S, 6.51.

*3,5*-*Dinitro*-*N*-*(pyrimidin*-*2*-*yl) benzamide***21**: Yield: (3.3 g, 53 %), m.p. 175 °C. IR (ν, cm^−1^): 3,307 (NH_str_), 3,091 (NH_str_), 2,961 (CH_str_ arom), 2,882, 2,677, 2,540, 1,854, 1,705, 1,627 (C=O_str_ amide), 1,543 (NO_2asymstr_), 1,470, 1,415, 1,350 (NO_2symstr_), 1,284 (C–N_str_ arom), 1,179, 1,078, 922, 799, 725, 695, 642, 532. Elemental analysis calculated for C_11_H_7_N_5_O_5_: C, 45.67; H, 2.42; N, 24.20. Found: C, 45.36; H, 2.73; N, 24.51.

*N*-*(4*-*Methylpyrimidin*-*2*-*yl)*-*3,5*-*dinitrobenzamide***22**: Yield: (3.4 g, 52 %), m.p. 200 °C. IR (ν, cm^−1^): 3,332 (NH_str_), 3,091 (NH_str_), 3,006 (CH_str_ arom), 2,962, 2,881 (CH_str_), 2,676, 2,539, 1,967, 1,852, 1,705, 1,628 (C=O_str_ amide), 1,600 (C=C_str_ arom), 1,544 (NO_2asymstr_), 1,470, 1,414, 1,350 (NO_2symstr_), 1,284 (C-N_str_ arom), 1,179, 1,076, 921, 807, 783, 725, 694, 643, 531. ^1^H NMR (500 MHz, DMSO) δ; 9.16 (1H, s, CONH), 9.11 (2H, s, H5 and H6 arom), 9.04 (1H, s, H2 arom), 8.17 (1H, d, pyrimidine H6^′^), 6.88 (1H, d, pyrimidine H5′), 2.43 (3H, s, CH_3_). Elemental analysis calculated for C_12_H_9_N_5_O_5_: C, 47.50; H, 2.97; N, 23.10. Found: C, 47.81; H, 2.63; N, 22.84.

*N*-*(4,6*-*Dimethylpyrimidin*-*2*-*yl)*-*3,5*-*dinitrobenzamide***23**: Yield: (3.5 g, 64 %), m.p. 168 °C. IR (ν, cm^−1^): 3,343 (NH_str_), 3,093(NH_str_), 3,028 (CH_str_ arom), 2,881 (CH_str_), 2,677, 2,540, 1,974, 1,701, 1,626 (C=O_str_ amide), 1,541(NO_2asym str_), 1,469, 1,417, 1,348 (NO_2symstr_), 1,285, 1,181, 1,077, 925, 834, 787, 724, 696, 634, 524. Elemental analysis calculated for C_13_H_11_N_5_O_5_: C, 49.21; H, 3.47; N, 22.08. Found: C, 49.48; H, 3.71; N, 21.92.

*3,5*-*Dinitro*-*N*-*(4*-*(N*-*pyrimidin*-*2*-*yl sulfamoyl) phenyl) benzamide***30**: Yield: (2.6 g, 68 %), m.p. 291 °C. IR (ν, cm^−1^): 3,399 (NH_str_ SO_2_NH), 3,102 (NH_str_ CONH), 3,040 (CH_str_ arom), 2,936, 2,868, 2,811, 2,732, 1,682 (C=O_str_ amide), 1,626, 1,587 (C=C_str_ arom), 1,537 (NO_2 str_), 1,492, 1,444, 1,406, 1,342 (SO_2 asymstr_), 1,266 (CN_str_ arom), 1,165 (SO_2 symstr_), 1,091, 1,001, 948, 921, 838, 798, 724, 680, 644, 570, 518. ^1^H-NMR (500 MHz, DMSO) δ 9.136 (s, 1H, CONH), 9.11 (s, 2H, H5, H6 arom), 8.8 (s, 1H, H2 arom), 8.47 (m, 2H, H4″ and H6″ arom), 7.98 (m, 2H, H2′ arom), 7.84 (m, 2H, H3′ arom), 7.02 (m, 1H, H5″ arom), 4.01 (s, 1H, SO_2_NH). MS-EI (m/z): 444 (4 *M*^+^), 410 (19), 407 (79), 361 (7), 304 (8), 277 (46), 249 (100), 232 (27), 205 (95), 152 (46), 140 (17), 127 (20), 77 (7), 29 (92). Elemental analysis calculated for C_17_H_12_N_6_O_7_S. DMF: C, 48.54; H, 4.89; N, 18.11; S, 5.18. Found: C, 48.06; H, 3.62; N, 18.07; S, 6.47.

*N*-*(4*-*(N*-*(4*-*Methylpyrimidin*-*2*-*yl) sulfamoyl) phenyl)*-*3,5*-*dinitrobenzamide***31**: Yield: (2.9 g, 73 %), m.p. 258 °C. IR (ν, cm^−1^): 3,453 (NH_str_ SO_2_NH), 3,401 (NH_str_ CONH), 3,100 (C–H_str_ arom), 2,859 (CH_str_), 2,776, 1,685 (C=O_str_ amide), 1,597, 1,541 (NO_2str_), 1,499, 1,446, 1,403, 1,345 (SO_2 asym str_), 1,289, 1,269 (CN_str_ arom), 1,242, 1,209, 1,159 (SO_2symstr_), 1,086, 966, 917, 890, 840, 795, 727, 677, 577. Elemental analysis calculated for C_18_H_14_N_6_O_7_S: C, 47.16; H, 3.06; N, 18.34; S, 6.98. Found: C, 47.43; H, 3.39; N, 18.67; S, 7.23.

*N*-*(4*-*(N*-*(4,6*-*Dimethylpyrimidin*-*2*-*yl) sulfamoyl) phenyl)*-*3,5*-*dinitrobenzamide***32**: Yield: (3.5 g, 57 %), m.p. 238 °C. IR (ν, cm^−1^): 3,438 (NH_str_ SO_2_NH), 3,111 (NH_str_ CONH), 2,921 (C–H_str_ arom), 2,854 (CH_str_), 1,682 (C=O_str_ amide), 1,628, 1,599 (C=C_str_ arom), 1,541 (NO_2str_), 1,436, 1,401, 1,345 (SO_2 asymstr_), 1,265 (CN_str_ arom), 1,159 (SO_2 sym str_), 1,141, 1,082, 1,031, 975, 919, 866, 842, 782, 715, 668, 587. Elemental analysis calculated for C_19_H_16_N_6_O_7_S: C, 48.30; H, 3.39; N, 17.79; S, 6.78. Found: C, 47.98; H, 2.89; N, 17.43; S, 7.02.

The above described 3,5-Dinitrobenzamide derivatives (3 g) dissolved in 30 ml of ethanol were treated with 100 mg of Pd/C (10 %). Hydrazine hydrate (15 ml) was added dropwise over a period of 1 h and the mixture was heated at 90 °C for 15 h. The catalyst was removed by filtration and the filtrate was concentrated under vacuum to dryness. The obtained solid was dried in a vacuum oven at 60 °C.

*3,5*-*Diamino*-*N*-*phenylbenzamide***13:** Yield: 80 %, m.p. 197 °C. IR (ν, cm^−1^): 3,437, 3,384 (NH_2 str_), 3,282 (NH_str_), 3,057 (CH_str_ arom), 3,015, 2,923, 1,652 (C=O_str_ amide), 1,595 (C = C_str_ arom), 1,532 (NH_2_), 1,498, 1,464, 1,435, 1,355 (CN_str_), 1,314, 1,255, 1,185, 1,029, 1,001, 855, 770, 749, 711, 683, 604, 564, 526, 476. ^1^H-NMR (500 MHz, DMSO) δ 9.14 (s, 1H, CONH), 6.99–7.702 (m, 5H, ArH), 6.45 (s, 2H, H5, H6 arom), 6.33 (s, 2H, NH_2_), 6.03 (s, 1H, H2 arom). Elemental analysis calculated for **8**; C_13_H_13_N_3_O: C, 68.72; H, 5.72; N, 18.50. Found: C, 68.44; H, 5.37; N, 18.28.

*N(2*-*Chlorophenyl)*-*3,5*-*diaminobenzamide***14**: Yield: 85 %, m.p. 155 °C. IR (ν, cm^−1^): 3,454, 3,415, 3,369 (NH_2str_), 3,250 (NH_str_), 3,081 (CH_str_ arom), 2,924, 1,680 (C=O_str_ amide), 1,641, 1,587 (C=C_str_ arom), 1,522 (NH_2_), 1,436, 1,348 (CN_str_), 1,231, 1,053, 996, 936, 873, 807, 751, 733, 659, 551, 518, 436. Elemental analysis calculated for **14**; C_13_H_12_ClN_3_O: C, 59.65; H, 4.59; N, 16.06. Found: C, 59.82; H, 4.76; N, 15.78.

*N(3*-*Chlorophenyl)*-*3,5*-*diaminobenzamide***15**: Yield: 91 %, m.p. 205 °C. IR (ν, cm^−1^): 3,480, 3,406, 3,362 (NH_2str_), 3,225 (NH_str_), 3,089 (CH_str_), 2,924, 1,674 (C=O_str_, amide), 1,634, 1,593 (CH_str_ arom), 1,520 (NH_2_), 1,477, 1,419, 1,347 (CN_str_), 1,274, 1,242, 1,107, 1,079, 996, 932, 905, 871, 815, 774, 730, 682, 598, 536, 437. Elemental analysis calculated for **15**; C_13_H_12_ClN_3_O: C, 59.65; H, 4.59; N, 16.06. Found: C, 59.42; H, 4.92; N, 16.39.

*N(4*-*Chlorophenyl)*-*3,5*-*diaminobenzamide***16**: Yield: 67 %, m.p. 139 °C. IR (ν, cm^−1^): 3,422, 3,394 (NH_2str_), 3,280 (NH_str_), 1,640 (C=O_str_ amide), 1,594 (CH_str_ arom), 1,526 (NH_2_), 1,491, 1,395, 1,353 (CN_str_), 1,309, 1,246, 1,190, 1,092, 1,010, 855, 710, 494, 428. ^1^H NMR (500 MHz, DMSO) δ; 9.15 (1H, s, CONH), 8.01 (2H, d, H2′ Ar–Cl), 7.69 (2H, d, H3′ Ar–Cl), 6.29 (2H, s, H5 and H6 arom), 6.29 (4H, s, NH_2_), 5.49 (1H, s, H2 arom). Elemental analysis calculated for **16**; C_13_H_12_ClN_3_O: C, 59.65; H, 4.59; N, 16.06. Found: C, 59.32; H, 4.86; N, 15.81.

*N(4*-*(N*-*(2*-*Chlorophenyl)sulfamoyl)phenyl)*-*3,5*-*diaminobenzamide***17**: Yield: 41 %, m.p. 154 °C. IR (ν, cm^−1^): 3,425, 3,360 (NH_2 str_), 1,670 (C=O_str_ amide), 1,592 (C = C_str_ arom), 1,518 (NH_2_), 1,483, 1,400, 1,324 (SO_2 asym str_), 1,229, 1,189, 1,156 (SO_2 symstr_), 1,094, 1,057, 999, 921, 868, 831, 760 (C–Cl_str_), 727, 682, 595, 565. Elemental analysis calculated for **17**; C_19_H_17_ClN_4_O_3_S: C, 54.74; H, 4.08; N, 13.44; S, 7.68. Found: C, 54.33; H, 4.41; N, 13.73; S, 7.39.

*3,5*-*Diamino*-*N*-*(pyrimidin*-*2*-*yl) benzamide***24**: Yield: 92 %, m.p. 200 °C. IR (ν, cm^−1^): 3,697, 3,453, 3,369 (NH_2str_), 3,086 (NH_str_), 2,924 (CH_str_ arom), 2,656, 1,631 (C=O_str_ amide), 1,572 (C=C_str_ arom), 1,526 (NH_2_), 1,463, 1,391, 1,340 (CN_str_), 1,295, 1,081, 999, 948, 917, 865, 797, 736, 657, 606, 544. Elemental analysis calculated for **24**; C_11_H_11_N_5_O: C, 57.64; H, 4.80; N, 30.57. Found: C, 57.34; H, 4.78; N, 30.86.

*N(4*-*Methylpyrimidin*-*2*-*yl)*-*3,5*-*diaminobenzamide***25**: Yield: 63 %, m.p. 118 °C. IR (ν, cm^−1^): 3,726, 3,340 (NH_2str_), 3,224 (NH_str_), 2,966 (CH_str_ arom), 2,629, 2,076, 1,626 (C=O_str_ amide), 1,567 (NH_2_), 1,469, 1,400 (CN_str_), 1,157, 998, 941, 859, 762, 674, 562, 498. ^1^HNMR (500 MHz, DMSO) δ; 9.15 (1H, s, CONH), 8.23 (1H, d, pyrimidine H6′), 6.88 (1H, d, pyrimidine H5′), 6.38 (2H, s, H5 and H6 arom), 6.25 (4H, s, NH_2_), 5.97 (1H, s, H2 arom), 2.391 (3H, s, CH_3_). Elemental analysis calculated for **25**; C_12_H_13_N_5_O: C, 59.26; H, 5.35; N, 28.80. Found: C, 59.63; H, 5.71; N, 29.03.

*N(4,6*-*Dimethylpyrimidin*-*2*-*yl)*-*3,5*-*diaminobenzamide***26**: Yield: 84 %, m.p. 186 °C. IR (ν, cm^−1^): 3,697, 3,459, 3,379 (NH_2 str_), 3,087 (NH_str_), 1,625 (C=O_str_ amide), 1,575 (C=C_str_ arom), 1,525 (NH_2_), 1,389, 1,337 (CN_str_), 1,081, 948, 882, 792, 735, 656, 606, 438. Elemental analysis calculated for **26**; C_13_H_15_N_5_O: C, 65.70; H, 5.83; N, 27.23. Found: C, 65.47; H, 5.52; N, 27.46.

*3,5*-*Diamino*-*N*-*(4*-*(N*-*pyrimidin*-*2*-*yl)sulfamoyl)phenyl)benzamide***33**: Yield: 46 %, m.p. 242 °C. IR (ν, cm^−1^): 3,448 (NH_str_ SO_2_NH), 3,422, 3,341 (NH_2str_), 3,204 (NH_str_ CONH), 1,635(C=O_str_ amide), 1,609 (CH_str_ arom), 1,523 (NH_2_), 1,395, 1,356 (SO_2 asymstr_), 1,311, 1,253 (CN_str_ arom), 1,165, 1,135 (SO_2symstr_), 1,087, 1,012, 822, 779, 756, 692, 633, 553. ^1^H-NMR (500 MHz, DMSO): δ 9.14 (s, 1H, CONH), 8.47 (m, 2H, H4″, H6″ arom), 7.96 (m, 2H H2″, ArH), 7.84 (m, 2H, H3′, ArH), 7.01 (m, 1H, H5″ arom), 6.48 (s, 2H, H5, H6 arom), 6.31 (s, 2H, NH_2_), 5.98 (s, 1H, H2 dinitro arom), 4.01 (s, 1H, SO_2_NH). Elemental analysis calculated for **33**; C_17_H_16_N_6_O_3_S: C, 53.12; H, 4.167; N, 21.87; S, 8.33. Found: C, 53.28; H, 4.36; N, 21.51; S, 8.12.

*N(4*-*(N*-*(4*-*Methylpyrimidin*-*2*-*yl)sulfamoyl)phenyl)*-*3,5*-*diaminobenzamide***34**: Yield: 80 %, m.p. 216 °C. IR (ν, cm^−1^): 3,731, 3,464 (NH_str_ SO_2_NH), 3,412, 3,343 (NH_2 str_), 3,276, 3,082 (NH_str_ CONH), 1,928, 1,815, 1,667 (C=O_str_ amide), 1,627, 1,590 (CH_str_ arom), 1,527 (NH_2_), 1,398, 1,332 (SO_2 asymstr_), 1,251 (CN_str_ arom), 1,192, 1,154 (SO_2 symstr_), 1,094, 873, 835, 688, 596, 545. Elemental analysis calculated for **34**; C_18_H_18_N_6_O_3_S: C, 54.27; H, 4.52; N, 21.10; S, 8.04. Found: C, 54.52; H, 4.86; N, 21.39; S, 8.40.

*N(4*-*(N*-*(4,6*-*Dimethylpyrimidin*-*2*-*yl) sulfamoyl) phenyl)*-*3,5*-*diaminobenzamide***35**: Yield: 40 %, m.p. 172 °C. IR (ν, cm^−1^): 3,561, 3,457 (NH_str_ SO_2_NH), 3,397, 3,361 (NH_2str_), 3,288 (NH_str_ CONH), 1,666 (C=O_str_ amide), 1,599 (C=C_str_ arom), 1,531 (NH_2_), 1,401, 1,368 (SO_2 asymstr_), 1,313 (CN_str_), 1,258 (CN_str_ arom), 1,186, 1,139 (SO_2symstr_), 1,079, 1,029, 962, 848, 780, 713, 677, 588, 548. Elemental analysis calculated for **35**; C_19_H_20_N_6_O_3_S: C, 55.34; H, 4.85; N, 20.39; S, 7.76. Found: C, 55.58; H, 5.09; N, 20.61; S, 7.99.

### Reaction of isophthaloyl chloride with 3,5-diaminobenzamide containing chloro aromatic pendant structures **13**–**17**: synthesis of polyamides **36**–**40** (General method)

The readily available isophthaloyl chloride (2.20 mmol) was slowly added to a stirred solution of the appropriate diamine **13**–**17** (2.20 mmol) dissolved in 10 ml DMA at 0 °C (ice bath). The mixture was stirred overnight at r.t then it was poured into iced water. The precipitate was collected by filtration, washed thoroughly with water, ethanol, and water again and dried in a vacuum oven at 80 °C.

#### Preparation of polymer **36**

Following the general method described above, isophthaloyl dichloride reacted with 3,5-diamino-N-phenylbenzamide **13** to give the polyamide **36**. The following data were recorded: Yield: 0.8 g, 97 %, m.p >300 °C, *η*_inh_ = 1.48 dL/g. IR (ν, cm^−1^): 3,727, 3,275 (NH_str_), 3,094 (CH_str_ arom), 2,924, 1,663 (C=O_str_ amide), 1,600 (C=C_str_ arom), 1,535, 1,441, 1.327, 1,239, 1,077, 1,010, 871, 820, 755, 715, 686, 585, 530. Elemental analysis calculated for the polyamide **36** (C_21_H_15_N_3_O_3_)_n_.H_2_O: C, 67.20; H, 4.53; N, 11.20. Found: C, 67.51; H, 4.78; N, 11.43.

#### Preparation of polymer **37**

Following the general method described above, isophthaloyl dichloride reacted *N*-(2-chloro phenyl)-3,5-diaminobenzamide **14** to give the polyamide **37**. The following data were recorded: Yield: 0.39 g, 50 %, m.p >300 °C, *η*_inh_ = 0.14 dL/g. IR (ν, cm^−1^): 3,387, 3,235 (NH_str_ amide), 3,081 (CH_str_ arom), 1,688, 1,660 (C=O_str_ amide), 1,590 (C=C_str_ arom), 1,532, 1,437, 1,343, 1,311, 1,237, 1,133, 1,085, 1,057, 947, 896, 817, 733 (C–Cl_str_), 688, 593, 551, 441. Elemental analysis calculated for polyamide **37** (C_21_H_14_ClN_3_O_3_)_n_.H_2_O: C, 61.50; H, 3.90; N, 10.26. Found: C, 61.22; H, 4.31; N, 10.59.

#### Preparation of polymer **38**

Following the general method described above, isophthaloyl dichloride reacted *N*-(3-chlorophenyl)-3,5-diaminobenzamide **15** to give the polyamide **38**; yield: 0.48 g, 62 %, m.p >300 °C, *η*_inh_ = 0.28 dL/g. IR (ν, cm^−1^): 3,291 (NH_str_ amide), 3,187, 3,081 (CH_str_ arom), 3,009, 2,883, 2,665, 2,553, 1,689, 1,661 (C=O_str_ amide), 1,593 (C=C_str_ arom), 1,533, 1,480, 1,421, 1,342, 1,285, 1,166, 1,080, 998, 893, 825 (C–Cl_str_), 780, 730, 685, 598, 539, 439. Elemental analysis calculated for polyamide **38** (C_21_H_14_ClN_3_O_3_)_n_.H_2_O: C, 61.50; H, 3.90; N, 10.26. Found: C, 61.79; H, 3.62; N, 10.54.

#### Preparation of polymer **39**

Following the general method described above, isophthaloyl dichloride reacted *N*-(4-chlorophenyl)-3,5-diaminobenzamide **16** to give the polyamide **39**; yield: 0.75 g, 95 %, m.p >300 °C, *η*_inh_ = 0.47 dL/g. IR (ν, cm^−1^): 3,298 (NH_str_ amide), 3,109 (CH_str_ aromatic), 1,661 (C=O_str_ amide), 1,599 (C=C_str_ arom), 1,535, 1,492, 1,445, 1,398, 1,330, 1,242, 1,090, 1,009, 874 (C–Cl_str_), 825, 719, 591, 504, 432. Elemental analysis calculated for polyamide **39** (C_21_H_14_ClN_3_O_3_)_n_. H_2_O: C, 61.50; H, 3.90; N, 10.26. Found: C, 61.83; H, 4.28; N, 10.61.

#### Preparation of polymer **40**

Following the general method described above, isophthaloyl dichloride reacted *N*-(4-(*N*-(2-chlorophenyl)sulfamoyl) phenyl)-3,5-diaminobenzamide **17** to give the polyamide **40**; yield: 0.65 g, 96 %, m.p >300 °C, *η*_inh_ = 0.89 dL/g. IR (ν, cm^−1^): 3,339 (NH_str_ amide), 3,105 (C–H_str_ arom), 2,925, 1,924, 1,665 (C=O_str_ amide), 1,594 (C=C_str_ arom), 1,528, 1,484, 1,446, 1,401, 1,330 (SO_2 asym str_), 1,250, 1,158 (SO_2 sym str_), 723, 686, 563. Elemental analysis calculated for polyamide **40** (C_27_H_19_ClN_4_O_5_S)_n_.H_2_O: C, 57.40; H, 3.72; N, 9.92; S, 5.67. Found: C, 57.79; H, 3.38; N, 9.56; S, 5.65.

### Reaction of pyridine 2,6-dicarbonyl dichloride with 3,5-diaminobenzamide containing chloroaromatic pendant structures **13**–**17**: synthesis of polyamides **41**–**45**

The readily available *pyridine 2,6*-*dicarbonyl dichloride* (2.20 mmol) was the appropriate diamine **13**-**17** (2.20 mmol) following the above mentioned general method.

#### Preparation of polymer **41**

Following the general method described above, *pyridine 2,6*-*dicarbonyl dichloride* reacted with 3,5-diamino-*N*-phenylbenzamide **13** to give the polyamide **41**. The following data were recorded: Yield: 0.8 g, 96 %, m.p >300 °C, η_inh_ = 1.44 dL/g. IR (ν, cm^−1^): 3,427 (NH_str_ amide), 2,968 (CH_str_ arom), 2,924, 1,675 (C=O_str_ amide), 1,600 (C=C_str_ arom), 1,531, 1,444, 1,328 (CN_str_ arom), 1,238, 1,184, 1,135, 1,076, 1,047, 997, 872, 749, 680, 530. Elemental analysis calculated for the polyamide **41** (C_20_H_14_N_4_O_3_)_n_.H_2_O: C, 63.80; H, 4.25; N, 14.89. Found: C, 63.52; H, 4.61; N, 14.51.

#### Preparation of polymer **42**

Following the general method described above, *pyridine 2,6*-*dicarbonyl dichloride* reacted with *N*-(2-chlorophenyl)-3,5-diaminobenzamide **14** to give the polyamide **42**; yield: 0.38 g, 48 %, m.p >300 °C, η_inh_ = 0.47 dL/g. IR (ν, cm^−1^): 3,785, 3,726, 3,698, 3,469, 3,406 (NH_str_ aromatic), 3,312 (NH_str_ amide), 2,926 (CH_str_ arom), 1,688, 1,623 (C=O_str_ amide), 1,591 (C=C_str_ arom), 1,532, 1,438, 1,346 (CN_str_ arom), 1,218, 1,135, 1,078, 1,034, 1,000, 942, 898, 845, 814,744 (C-Cl_str_), 680, 555. Elemental analysis calculated for polyamide **42** (C_20_H_13_ClN_4_O_3_)_n_.H_2_O: C, 58.46; H, 3.65; N, 13.64. Found: C, 58.76; H, 3.34; N, 13.25.

#### Preparation of polymer **43**

Following the general method described above, *pyridine 2,6*-*dicarbonyl dichloride* reacted with *N*-(3-chlorophenyl)-3,5-diaminobenzamide **15** to give the polyamide **43**; yield: 0.37 g, 47 %, m.p >300 °C, η_inh_ = 0.52 dL/g. IR (ν, cm^−1^): 3,728, 3,427 (NH_str_ arom), 3,298 (NH_str_ amide), 3,107 (CH_str_ arom), 1,677 (C=O_str_ amide), 1,599 (C=C_str_ arom), 1,533, 1,493, 1,449, 1,397, 1,310 (CN_str_ arom), 1,239, 1,190, 1,142, 1,084, 1,004, 942, 873 (C–Cl_str_), 828, 748, 678, 504. Elemental analysis calculated for the polyamide **43** (C_20_H_13_ClN_4_O_3_)_n_.H_2_O: C, 58.46; H, 3.65; N, 13.64. Found: C, 58.71; H, 3.89; N, 13.27.

#### Preparation of polymer **44**

Following the general method described above, *pyridine 2,6*-*dicarbonyl dichloride* reacted with *N*-(4-chlorophenyl)-3,5-diaminobenzamide **16** to give the polyamide **44**; yield: 0.67 g, 85 %, m.p >300 °C, η_inh_ = 0.72 dL/g. IR (ν, cm^−1^): 3,728, 3,298 (NH_str_ amide), 3,106 (C–H_str_ arom), 2,117, 1,988, 1,678 (C=O_str_ amide), 1,599 (C=C_str_ arom), 1,533, 1,493, 1,449, 1,397, 1,310 (CN_str_ arom), 1,238, 1,189, 1,142, 1,085, 1,004, 942, 872, 828 (C–Cl_str_), 748, 678, 504. Elemental analysis calculated for the polyamide **44** (C_20_H_13_ClN_4_O_3_)_n_.H_2_O: C, 58.46; H, 3.65; N, 13.64. Found: C, 58.18; H, 3.26; N, 13.32.

#### Preparation of polymer **45**

Following the general method described above, *pyridine 2,6*-*dicarbonyl dichloride* reacted with *N*-(4-(*N*-(2-chlorophenyl) sulfamoyl) phenyl)-3,5-diaminobenzamide **17** to give the polyamide **45**; yield: 0.66 g, 97 %, m.p >300 °C, η_inh_ = 0.75 dL/g. IR (ν, cm^−1^): 3,266 (N–H_str_ amide), 3,105 (CH_str_ arom), 2,396, 1,781, 1,682 (C=O_str_ amide), 1,595 (C=C_str_ arom), 1,527, 1,485, 1,452, 1,400, 1,330 (SO_2asymstr_), 1,253, 1,222, 1,159 (SO_2symstr_), 1,091, 1,056, 1,000, 916, 837, 752, 725 (C–Cl_str_), 683, 628, 562. Elemental analysis calculated for the polyamide **45** (C_26_H_18_ClN_5_O_5_S)_n_.H_2_O: C, 55.17; H, 3.50; N, 12.38; S, 5.66. Found: C, 55.49; H, 3.82; N, 12.59; S, 5.94.

### Reaction of isophthaloyl chloride with 3,5-diaminobenzamide containing substituted pyrimidine-2-yl pendant structures **24**–**26**: synthesis of polyamides **46**–**48** (general method)

Isophthaloyl chloride (2.20 mmol) was treated with a solution of the appropriate diamine **24**–**26** (2.20 mmol) following the general procedure described above.

#### Preparation of polymer **46**

Following the general method, isophthaloyl dichloride was treated with 3,5-diamino-N-(pyrimidin-2-yl)benzamide **24** to furnish the polymer **46**; yield: 0.66 g, 88 %, m.p >300 °C, *η*_inh_ = 0.80 dL/g. IR (ν, cm^−1^): 3,399 (NH_str_ amide), 2,924 (CH_str_ arom), 1,655 (C=O_str_ amide), 1,536 (C=C_str_ arom), 1,346, 1,267, 1,086, 904, 784, 733, 682, 594. Elemental analysis calculated for the polyamide **46** (C_19_H_13_N_5_O_3_)_n_.H_2_O: C, 60.48; H, 3.98; N, 18.56. Found: C, 60.18; H, 3.61; N, 18.22.

#### Preparation of polymer **47**

Following the general method, isophthaloyl dichloride was treated with *N*-(4-methylpyrimidin -2-yl)-3,5-diaminobenzamide **25** to furnish the polymer **47**; yield: 0.7 g, 88 %, m.p >300 °C, *η*_inh_ = 1.16 dL/g. IR (ν, cm^−1^): 3,432 (NH_str_ amide), 2,924 (CH_str_ arom), 1,657 (C=O_str_ amide), 1,610 (C=C_str_ arom), 1,543, 1,448, 1,333, 1,231, 876, 772, 713, 597. Elemental analysis calculated for the polyamide **47** (C_20_H_15_N_5_O_3_)_n_.H_2_O: C, 61.38; H, 4.35; N, 17.90. Found: C, 61.64; H, 4.58; N, 17.52.

#### Preparation of polymer **48**

Following the general method, isophthaloyl dichloride was treated with *N*-(4,6-dimethyl pyrimidin-2-yl)-3,5-diaminobenzamide **26** to furnish the polymer **48**; yield: 0.65 g, 83 %, m.p >300 °C, *η*_inh_ = 0.96 dL/g. IR (ν, cm^−1^): 3,396 (NH_str_ amide), 2,924 (CH_str_ arom), 1,658 (C=O_str_ amide), 1,536 (C=C_str_ arom), 1,435, 1,346, 1,268 (CN_str_ arom), 1,087, 905, 785, 733, 682, 596. Elemental analysis calculated for the polyamide **48** (C_21_H_17_N_5_O_3_)_n_.H_2_O: C, 62.20; H, 4.69; N, 17.28. Found: C, 62.60; H, 4.24; N, 17.57.

### Reaction of pyridine 2,6-dicarbonyl dichloride with 3,5-diaminobenzamide containing substituted pyrimidine-2-yl pendant structures **24**–**26**: synthesis of polyamides **49**-**51**

Pyridine 2,6-dicarbonyl dichloride (2.20 mmol) was treated with a solution of the appropriate diamine **24**–**26** (2.20 mmol) in DMA as described earlier.

#### Preparation of polymer **49**

Following the described general method, pyridine 2,6-dicarbonyl dichloride was reacted with 3,5-diamino-*N*-(pyrimidin-2-yl) benzamide **24** to produce the polymer **49**; yield: 0.74 g, 80 %, m.p >300 °C, η_inh_ = 0.32 dL/g. IR (ν, cm^−1^): 3,438 (NH_str_ amide), 2,923 (CH_str_ arom), 2,856, 1,696, 1,666 (C=O_str_ amide), 1,629 (C=N_str_ arom), 1,595 (C=C_str_ arom), 1,536, 1,436, 1,346 (C–N_str_ arom), 1,278, 1,222, 1,136, 1,078, 1,000, 906, 843, 784, 742, 658, 601. Elemental analysis calculated for polyamide **49** (C_18_H_12_N_6_O_3_)_n_.H_2_O: C, 57.14; H, 3.70; N, 22.20. Found: C, 57.48; H, 3.41; N, 22.52.

#### Preparation of polymer **50**

Following the described method, pyridine-2,6-dicarbonyl dichloride was treated with N-(4-methylpyrimidin-2-yl)-3,5-diaminobenzamide **25** to give the polymer **50**; yield: 0.8 g, 83 %, m.p >300 °C, η_inh_ = 0.54 dL/g. IR (ν, cm^−1^): 3,726, 3,435 (NH_str_ amide), 2,924 (CH_str_ arom), 2,858 (CH_str_), 1,681 (C=O_str_ amide), 1,607 (C=C_str_ arom), 1,539, 1,453, 1,332 (CN_str_ arom), 1,297, 1,221, 1,139, 1,078, 997, 873, 775, 743, 670, 617. Elemental analysis calculated for polyamide **50** (C_19_H_14_N_6_O_3_)_n_.H_2_O: C, 58.16; H, 4.08; N, 21.43. Found: C, 58.41; H, 4.39; N, 21.16.

#### Preparation of polymer **51**

Following the method described earlier, pyridine-2,6-dicarbonyl dichloride was reacted with *N*-(4,6-dimethylpyrimidin-2-yl)-3,5-diaminobenzamide **26** to give the polymer **51**; yield: 0.75 g, 76 %, m.p >300 °C, η_inh_ = 0.28 dL/g. IR (ν, cm^−1^): 3,444 (NH_str_ arom), 3,294 (NH_str_ amide), 3,093 (CH_str_ arom), 2,926, 2,860 (CH_str_), 1,695, 1,667 (C=O_str_ amide), 1,595 (C=C_str_ arom), 1,537, 1,434, 1,347 (CN_str_ arom), 1,281, 1,223, 1,139, 1,079, 1,000, 906, 843, 786, 743, 660, 601. Elemental analysis calculated for the polyamide **51** (C_20_H_16_N_6_O_3_)_n_.H_2_O: C, 59.10; H, 4.43; N, 20.69. Found: C, 59.38; H, 4.71; N, 20.37.

### Reaction of isophthaloyl chloride with 3,5-diaminobenzamide containing substituted sulfonamidopyrimidine-2-yl pendant structures **33**–**35**: synthesis of polyamides **52**–**54**

Isophthaloyl chloride (2.20 mmol) was reacted with the appropriate diamine **33**–**35** (2.20 mmol) following the general method described above.

#### Preparation of polymer **52**

Following the general method described above, isophthaloyl dichloride was reacted with 3,5-diamino-*N*-(4-(*N*-pyrimidin-2-ylsulfamoyl)phenyl)benzamide **33** to give the polymer **52**; yield: 1.2 g, 91 %, m.p >300 °C, η_inh_ = 1.16 dL/g. IR (ν, cm^−1^): 3,435 (NH_str_ amide), 2,924 (CH_str_ arom), 1,659 (C=O_str_ amide), 1,597 (C=C_str_ arom), 1,531, 1,443, 1,326 (SO_2asymstr_), 1,249, 1,154 (SO_2symstr_), 899, 830, 683, 593, 546. Elemental analysis calculated for the polyamide **52** (C_25_H_18_N_6_O_5_S)_n_.H_2_O: C, 56.39; H, 3.76; N, 15.79; S, 6.01. Found: C, 56.52; H, 3.44; N, 15.63; S, 6.34.

#### Preparation of polymer **53**

Following the same described method, isophthaloyl dichloride was treated with *N*-(4-(*N*-(4-methylpyrimidin-2-yl) sulfamoyl) phenyl)-3,5-diaminobenzamide **34** to furnish the polymer **53**; yield: 1 g, 80 %, m.p >300 °C, η_inh_ = 0.70 dL/g. IR (ν, cm^−1^): 3,404 (NH_str_ amide), 2,924 (CH_str_ arom), 1,665 (C=O_str_ amide), 1,596 (C=C_str_ arom), 1,531, 1,437, 1,400, 1,327 (SO_2asymstr_), 1,247, 1,154 (SO_2symstr_), 1,094, 898, 831, 684, 592, 546. Elemental analysis calculated for the polyamide **53** (C_26_H_20_N_6_O_5_S)_n_.H_2_O: C, 57.14; H, 4.03; N, 15.38; S, 5.86. Found: C, 57.48; H, 4.36; N, 15.66; S, 6.09.

#### Preparation of polymer **54**

Following the same described method given above, isophthaloyl dichloride was reacted with *N*-(4-(*N*-(4,6-dimethylpyrimidin-2-yl)sulfamoyl)phenyl)-3,5-diaminobenzamide **35** to produce the polymer **54**; yield: 0.6 g, 98 %, m.p >300 °C, η_inh_ = 0.81 dL/g. IR (ν, cm^−1^): 3,432 (NH_str_ amide), 2,924 (CH_str_ arom), 1,678 (C=O_str_ amide), 1,599 (C=C_str_ arom), 1,534, 1,441, 1,301 (SO_2asymstr_), 1,259, 1,154 (SO_2symstr_), 1,081, 862, 723, 685, 587. Elemental analysis calculated for the polyamide **43** (C_27_H_22_N_6_O_5_S)_n_.H_2_O: C, 57.86; H, 4.28; N, 15.00; S, 5.71. Found: C, 57.64; H, 4.59; N, 14.72; S, 5.43.

### Reaction of pyridine 2,6-dicarbonyl dichloride with 3,5-diaminobenzamide containing substituted sulfonamidopyrimidine-2-yl pendant structures **33**–**35**: synthesis of polyamides **55**–**57**

Pyridine 2,6-dicarbonyl dichloride (2.20 mmol) was treated with s solution of the appropriate diamine **22**–**24** in DMA as described earlier.

#### Preparation of polymer **55**

Pyridine 2,6-dicarbonyl dichloride was treated with 3,5-diamino-*N*-(4-(*N*-pyrimidin-2-yl sulfamoyl) phenyl)benzamide **33** following the described method mentioned earlier to give the polymer **55**; yield: 1.24 g, 95 %, m.p >300 °C, η_inh_ = 0.24 dL/g. IR (ν, cm^−1^): 3,726, 3,435 (NH_str_ amide), 2,923 (CH_str_ arom), 2,856, 1,668 (C=O_str_ amide), 1,595 (C=C_str_ arom), 1,528, 1,447, 1,398, 1,321 (SO_2asymstr_), 1,247, 1,153 (SO_2symstr_), 1,091, 888, 834, 748, 681, 590. Elemental analysis calculated for the polyamide **55** (C_24_H_17_N_7_O_5_S)_n_.H_2_O: C, 54.03; H, 3.56; N, 18.39; S, 6.00. Found: C, 54.32; H, 3.81; N, 18.65; S, 6.21.

#### Preparation of polymer **56**

Pyridine-2,6-dicarbonyl dichloride was reacted with *N*-(4-(*N*-(4-methylpyrimidin-2-yl)sulfamoyl) phenyl)-3,5-diaminobenzamide **34** to give the polymer **56**; yield: 1.2 g, 89 %, m.p >300 °C, η_inh_ = 0.35 dL/g. IR (ν, cm^−1^): 3,726, 3,437 (NH_str_ amide), 2,924 (CH_str_ arom), 2,857 (CH_str_), 1,678 (C=O_str_ amide), 1,595 (C=C_str_ arom), 1,529, 1,449, 1,398, 1,322 (SO_2asymstr_), 1,248, 1,153 (SO_2symstr_), 1,089, 1,004, 895, 834, 739, 680, 593. Elemental analysis calculated for polyamide **56** (C_25_H_19_N_7_O_5_S)_n_.H_2_O: C, 54.84; H, 3.84; N, 17.91; S, 5.85. Found: C, 55.09; H, 4.17; N, 17.62; S, 6.19.

#### Preparation of polymer **57**

Pyridine-2,6-dicarbonyl dichloride was treated with *N*-(4-(*N*-(4,6-dimethylpyrimidin-2-yl) sulfamoyl) phenyl)-3,5-diaminobenzamide **35** to give the polymer 46; yield: 1.2 g, 87 %, m.p >300 °C, *η*_inh_ = 0.55 dL/g. IR (ν, cm^−1^): 3,440 (NH_str_ amide), 2,923 (CH_str_ arom), 2,856 (CH_str_), 1,680 (C=O_str_ amide), 1,597 (C=C_str_ arom), 1,530, 1,444, 1,309 (SO_2asymstr_), 1,249, 1,151 (SO_2 asymstr_), 1,077, 1,003, 841, 787, 747, 679, 585. Elemental analysis calculated for polyamide **57** (C_26_H_21_N_7_O_5_S)_n_.H_2_O: C, 55.61; H, 4.10; N, 17.47; S, 5.70. Found: C, 55.90; H, 4.36; N 17.73; S, 5.34.

### Polymer particles synthesis (general Method)

The appropriate, readily available isophthaloyl dichloride, pyridine 2,6-dicarbonyl dichloride or the prepared one **60** (0.5 mmol) and the appropriate diamine **13**–**17**, **24**–**26**, **33**–**35** or **61**–**66** (0.5 mmol) were separately dissolved in dioxane (50 mL). Distilled water (10 mL) was added to the diamine and the entire solution was added to the acid chloride. The resulted turbid solution was ultrasonicated at 42 kHz in a water bath for a period of 30 min. The polymer colloidal solution was extracted by centrifugal separation for 15 min. at 6,000 rpm and the resulted precipitate were carefully washed with methanol and water to purify the product of any unreacted monomer. The polymer samples were then dried in a vacuum oven at 60 °C for 10 h.

## Results and discussions

### Synthesis of 3,5-diaminobenzamide containing pendent aromatic structures **8**–**12**, **16**–**18** and **22**–**24**

**Scheme 1:**
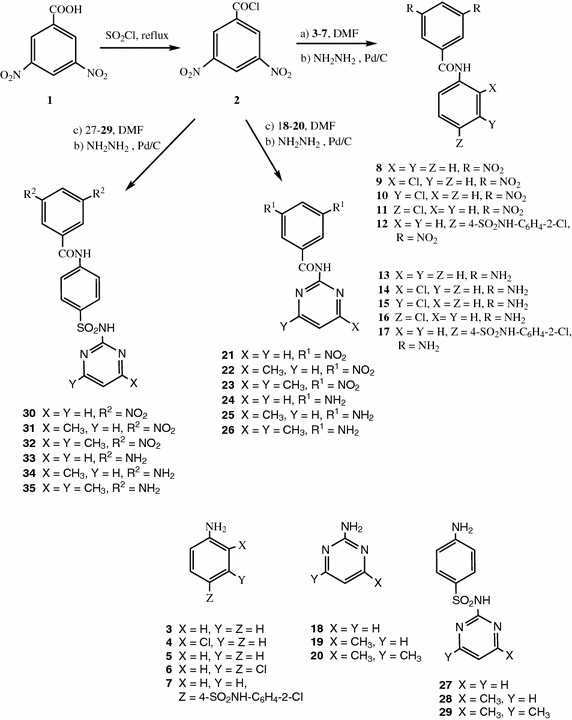
Synthesis of 3,5-diaminobenzamides containing aromatic pendent structures **8**–**17**, **21**–**26** and **30**–**35**

3,5-Diaminobenzamide containing pendent chloro aromatic structures **18**–**12**, Scheme 1, were prepared by the reaction of 3,5-dinitrobenzoic acid **1** with thionyl chloride and the obtained acid chloride **2** was then subsequently treated with a number of commercial substituted anilines **3**–**6** or 4-amino-*N*-(2-chlorophenyl) benzenesulfonamide **7**, respectively, in DMF to furnish the corresponding substituted 3,5-dinitrobenzamide **8**–**12**. The FT-IR spectra exhibited bands in the region 3,253–3,361 cm^−1^ belong to the NH_str_ absorption, a band at 1,650 cm^−1^ due to C=O amide while the NO_2_ bands appeared at 1,541 and 1,340 cm^−1^, the SO_2_ appeared at 1,339 and 1,158 cm^−1^. The reaction of the dinitrobenzamides **8**–**12** with hydrazine hydrate/Pd–C (10 %) furnished the corresponding 3,5-diaminobenzamides **13**–**17**, respectively, in good yields. The FT-IR spectra of diamines **13–17** showed absorption bands correspond to NH_2_ in the region 3,433–3,454 cm^−1^ and 3,360–3,394 cm^−1^, a characteristic band at 3,250 cm^−1^ due to the NH while the C=O bands were in region 1,640–1,680 cm^−1^.

Similarly, 3,5-diaminobenzamide containing pendent pyrimidine or sulfonamide pyrimidine **24**–**26** and **33**–**35** were prepared by reaction the acid chloride **2** with the amines **18**–**20**; namely: 2-aminopyrimidine **18**, 2-amino-4-methylpyrimidine **19**, 2-amino-4,6-dimethylpyrimidine **20** or aminosulfonamides **27**-**29** namely; sulfadiazine **27**, sulfamerazine **28** and sulfamethazine **29**, respectively, in DMF. The IR spectra of the prepared dinitro compounds **21**–**23** and **30**–**33** exhibited bands at 3,100 cm^−1^ correspond to the amide NH; bands in the region 1,626–1,685 cm^−1^ due to C=O amide, while the NO_2_ bands appeared at 1,540 cm^−1^. The obtained 3,5-dinitrobenzamides were reduced using hydrazine hydrate/Pd–C (10 %) mixture following standard procedure. The IR spectra of the diamines **24**–**26** and **33**–**35** showed absorption bands correspond to the NH_2_ at 3,453 and 3,361 cm^−1^; bands around 3,204 cm^−1^ due to the carbonyl NH and the C=O amide bands were in region 1,625–1,666 cm^−1^. Physical properties of all new compounds are recorded in the experimental section and the calculated analysis data are in good agreement with the experimental one.

### Synthesis of polyamides containing pendent chloro aromatic and pyrimidine- and sulfonamidopyrimidine pendent structures **36–45**, **46–51**, **52–57**

**Fig. 1:**
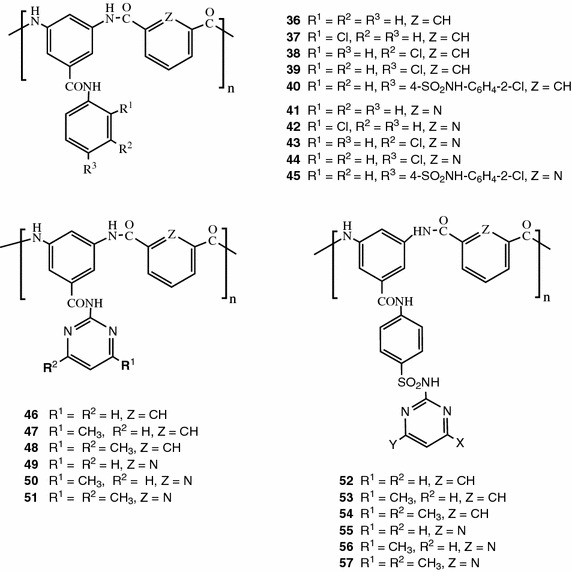
Chemical structures of the polyamides **36**–**45**, **46–51** and **52–57**

The production of new aromatic polyamides containing chloro aromatic, pyrimidine- and sulfonamidopyrimidine pendent structures, where the pendent groups act as signaling units due to their fluorescent, chromogenic and biological characteristics and studying of their properties are the objectives of our study. The important tasks in this study are to analyze and predict the properties such as solubility, optical and fluorescence emission properties, biological activities and thermal stability with respect to their chemical structures. The targeted polyamides **36**–**45**, **46–51**, **52–57** were synthesized in bulk scale by direct polycondensation of an equimolar mixture of the readily available isophthaloyl dichloride or pyridine-2,6-dicarbonyl dichloride with, respectively, the diamines **13**–**17**, **24**–**26**, **33**–**35** in DMA solutions at 0 °C (ice bath), Fig. 1. The polyamides were obtained in moderate to good yields and their inherent viscosities (η_inh_) are in the range of 0.24–1.48 dL/g. Physical properties of the new compounds are recorded in the experimental section and the calculated analysis data are in good agreement with the experimental one.

**Fig. 2:**
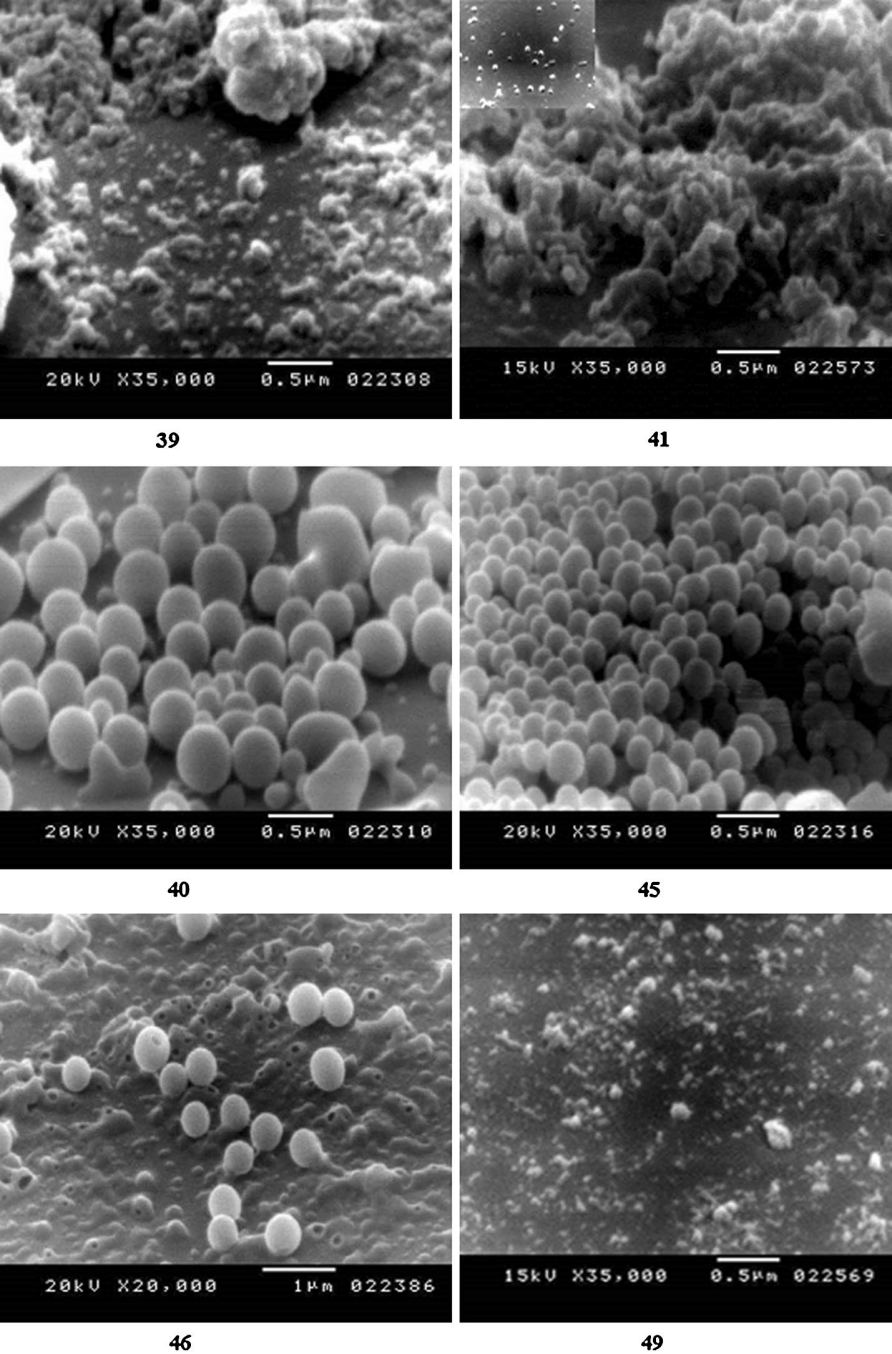
SEM images of the nanosized aramides **39–41**, **45–46** and **49**

**Fig. 3:**
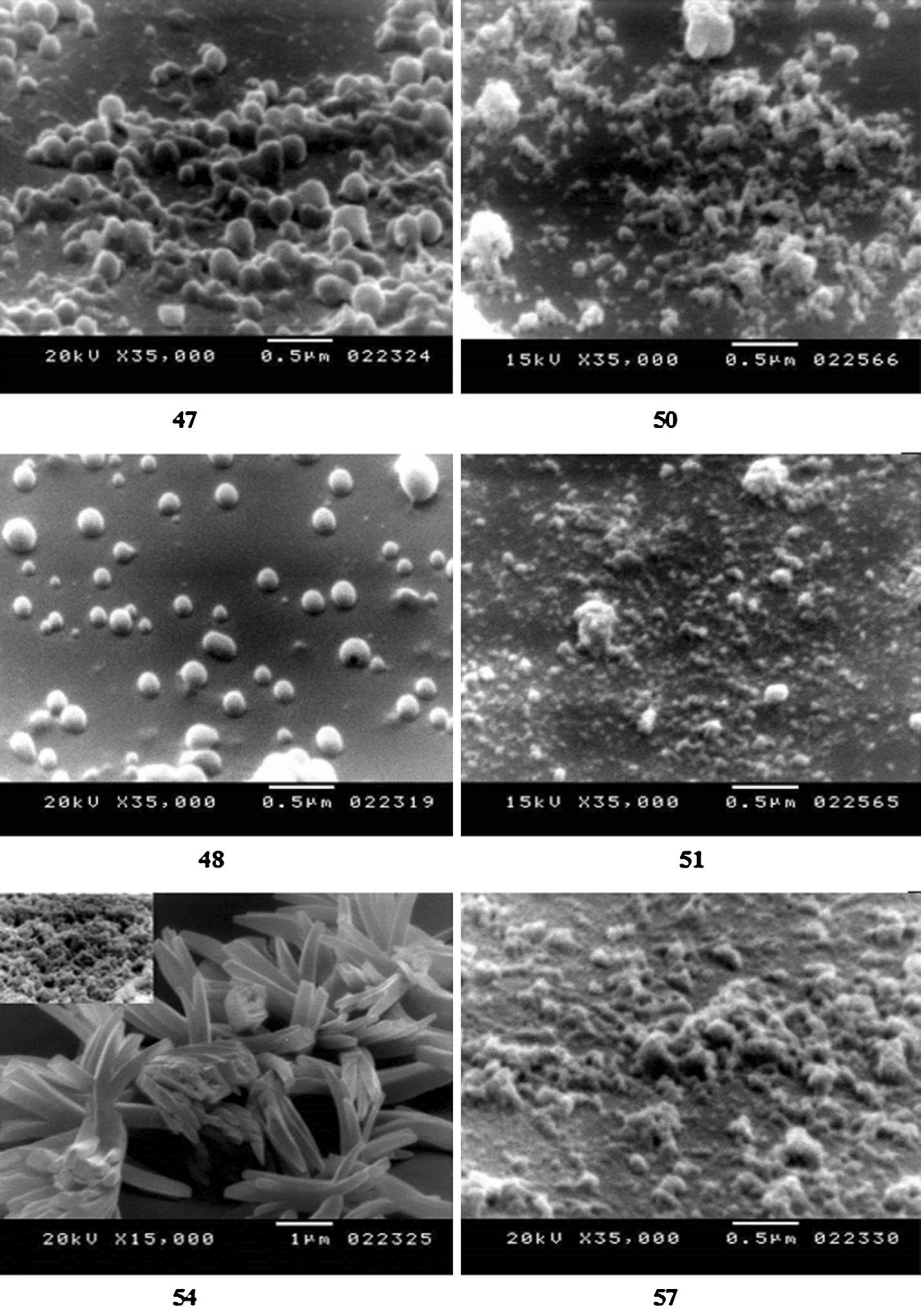
SEM images of the nanosized aramides **47**–**48**, **50–51**, **54** and **57**

The synthesis of the nanosized aramides particles **36–45**, **46–51** and **52–57** was the next task. Different solution techniques are known in the literature for the preparation of nanosized particles, including emulsion, interfacial polycondensations or nanoprecipitation method [23–29]. The basic principle of the latter method is based on the interfacial deposition of a polymer from solvent/non-solvent phases. Generally, the current series were prepared by ultrasonication of 0.5 mmol of the appropriate diamine with 0.5 mmol of the acid chloride in a total of 115 ml dioxane solution containing distilled water (15 ml) followed by centrifugal separation at 6,000 rpm for 30 min. The presence of water is necessary for controlling the particle shape and as a reaction accelerator. As judged by SEM micrographs, Figs. 2 and 3, most polyamides were obtained as well-separated spherical nanosized forms, nevertheless, there were some degree of aggregation for those polymers containing pyridine and pyrimidine pendant groups. The aggregate formation could be attributed to the molecular H-bond self-assembly via H-bond directed organization of molecular precursors. The average diameters (standard deviation) of some polymers were **39**; 66.76 nm (28.36), **40**; 198.86 (27.45), **41** 92.31 nm (27.59) and **45**; 209.27 nm (10.63); **46**; 406.12 nm (39.12), **47**; 205.6 nm (34.31), **48**; 77.27 nm (25.6), **49**; 48.29 nm (9.8), **50**; 58.99 (13.37), **51**; 61.08 nm (5.44); **54**; 69.6 nm (13.43) and **57**; 71.02 nm (18.85), respectively.

### Physical properties of the polymers

#### Solubility

The polyamides are readily soluble in polar aprotic solvents such as NMP, DMAc, DMF and DMSO while insoluble in boiling alcoholic or halogenated solvents. The observed solubility of the pyridine-containing polyamide compared to that polyamides containing phenylene moiety could be attributed to the dipole–dipole interaction of polymer–solvent system. The pyrimidine-containing polymers showed inferior (lower) solubility may be due to the presence of pyrimidine structural that aggravate macromolecule hydrogen. The presence of the sulfonamide group leads to increase solubility due to their effective contribution to the cohesive energy which counteracting their influence in the increment in the main chain–main chain distance.

#### Inherent viscosity

The inherent viscosity (η_inh_) of the polymers, as a suitable criterion for evaluation of molecular weight, was measured at a concentration of 0.5 g/100 mL in DMSO at 30 °C. The η_inh_ of phenylene-containing polyamides **36–40** were in the range 0.14–1.48 dL/g while their analogues **41–45** were in the range 0.47–1.44 dL/g indicating low to moderate molecular weights in this series. The η_inh_ of the amido- and sulfonamido-pyrimidine containing polymers **46–51**, **52–57**, respectively, were closely similar in the range 0.24–1.61 dL/g. Noteworthy, no significant change in inherent viscosity was noticed on phenylene/pyridine replacement.

#### FT-IR spectra

In general, the IR spectra of the phenylene-containing dinitro derivatives **8–12** exhibited bands in the region 3,253–3,361 cm^−1^ correspond to the NH_str_; a characteristic band at 1,650 cm^−1^ due to the C=O_str_ amide while the NO_2_ appeared at 1,541 cm^−1^ (–NO_2asymstr_) and 1,340 cm^−1^ (–NO_2symstr_). The SO_2_ sulfonamide in **12** appeared at 1,339 cm^−1^ (–SO_2asymstr_), 1,158 cm^−1^ and (–SO_2symstr_). Pyrimidine-containing dinitro derivatives **21**–**23** and **30**–**32** exhibited absorption bands at 3,100 cm^−1^ attributed to the NH_str_; a characteristic band in the region 1,626–1,685 cm^−1^ correspond to the C=O_str_ amide while the NO_2_ bands appeared at 1,540 cm^−1^.

The IR analyses of the phenylene- and pyrimidine-containing diamines **13**–**17**, **24**–**26** and **33**–**35** showed absorption bands in the region 3,433–3,454 cm^−1^ correspond to the amino groups (–NH_2asymstr_) and 3,360–3,394 cm^−1^ due to (–NH_2symstr_); the band at 3,250 cm^−1^ due to the NH_str_ band while the C=O_str_ amide bands were in region 1,640–1,680 cm^−1^. The SO_2_ sulfonamide bands in the diamines **33**–**35** appeared at 1,345 cm^−1^ (–SO_2asymstr_) and 1,159 cm^−1^ (–SO_2symstr_).

The IR analyses of the phenylene-containing polymers **36**–**40** exhibited major absorption bands, respectively, **36**: 3,275 (NH_str_), 3,094 (CH_str_ arom), 1,663 (C=O_str_ amide), 1,600 (C=C_str_ arom); **37**: 3,387, 3,235 (NH_str_ amide), 3,081 (CH_str_ arom), 1,688, 1,660 (C=O_str_ amide), 1,590 (C=C_str_ arom), 733 (CCl_str_); **38**: 3,291 (NH_str_ amide), 3,081 (CH_str_ arom), 1,689, 1,661 (C=O_str_ amide), 1,593 (C=C_str_), 825 (CCl_str_); **39**: 3,298 (NH_str_ amide), 3,109 (CH_str_ arom), 1,661 (C=O_str_ amide), 1,599 (C=C_str_ arom), 874 (C–Cl_str_); **40**: 3,339 (NH_str_ amide), 3,105 (CH_str_ arom), 1,924, 1,665 (C=O_str_ amide), 1,594 (C=C_str_ arom), 1,330 (SO_2asymstr_), 1,250, 1,158 (SO_2symstr_).

The IR analyses of the pyridine-containing polymers **41**–**45** showed major absorption bands, respectively, **41**: 3,427 (NH_str_ amide), 2,968 (CH_str_ arom), 1,675 (C=O_str_ amide), 1,600 (C=C_str_ arom), 1,328 (CN_str_ arom); **42**: 3,406 (NH_str_), 3,312 (NH_str_ amide), 1,688, 1,623 (C=O_str_ amide), 1,591 (C=C_str_ arom), 1,346 (CN_str_ arom), 744 (C–Cl_str_); **43**: 3,427 (NH_str_), 3,298 (NH_str_ amide), 3,107 (CH_str_ arom), 1,677 (C=O_str_ amide), 1,599 (C=C_str_ arom), 1,310 (CN_str_), 873 (C–Cl_str_); **44**: 3,728, 3,298 (NH_str_ amide), 3,106 (CH_str_ arom), 1,678 (C=O_str_ amide), 1,599 (C=C_str_ arom), 828 (C–Cl_str_); **45**: 3,266 (NH_str_ amide), 3,105 (CH_str_ arom), 1,682 (C=O_str_ amide), 1,595 (C=C_str_ arom), 1,330 (SO_2asymstr_), 1,159 (SO_2symstr_), 725 (C–Cl_str_).

The IR spectral data of the pyrimidineamido-containing polymers **46**–**51** exhibited absorption bands, respectively, **46**: 3,399 (NH_str_ amide), 1,655 (C=O_str_ amide), 1,536 (C=C_str_ arom); **47**: 3,432 (NH_str_ amide), 1,657 (C=O_str_ amide), 1,610 (C=C_str_ arom); **48**: 3,396 (NH_str_ amide), 1,658 (C=O_str_ amide), 1,536 (C=C_str_ arom); **49**: 3,438 (NH_str_ amide), 1,696, 1,666 (C=O_str_ amide), 1,629 (C=N_str_ arom), 1,595 (C=C_str_ arom); **50**: 3,435 (NH_str_ amide), 1,681 (C=O_str_ amide), 1,607 (C=C_str_ arom); **51**: 3,444 (NH_str_ arom), 3,294 (NH_str_ amide), 3,093 (CH_str_ arom), 1,695, 1,667 (C=O_str_ amide), 1,595 (C=C_str_ arom).

The IR spectral data of the pyrimidinesulfonamido-containing polymers **52**–**57** showed absorption bands, respectively, **52**: 3,435 (NH_str_ amide), 1,659 (C=O_str_ amide), 1,597 (C=C_str_ arom), 1,326 (SO_2asymstr_), 1,154 (SO_2symstr_); **53**: 3,404 (NH_str_ amide), 1,665 (C=O_str_ amide), 1,596 (C=C_str_ arom), 1,327 (SO_2asymstr_), 1,154 (SO_2symstr_); **54**: 3,432 (NH_str_ amide), 1,678 (C=O_str_ amide), 1,599 (C=C_str_ arom), 1,301 (SO_2asymstr_), 1,154 (SO_2symstr_); **55**: 3,435 (NH_str_ amide), 1,668 (C=O_str_ amide), 1,595 (C=C_str_ arom), 1,321 (SO_2asymstr_), 1,153 (SO_2symstr_); **56**: 3,437 (NH_str_ amide), 1,678 (C=O_str_ amide), 1,595 (C=C_str_ arom), 1,322 (SO_2asymstr_), 1,153 (SO_2symstr_); **57**: 3,440 (NH_str_ amide), 1,680 (C=O_str_ amide), 1,597 (C=C_str_ arom), 1309 (SO_2asymstr_), 1,151 (SO_2 asymstr_).

#### Optical properties

##### The optical properties of the polyamides **36–45**

The optical properties of polyamides series containing chloroaromatic pendent moiety and **36**–**45** were investigated by UV–vis and photoluminescence spectroscopy in DMSO using concentration of 2 mg/10 ml. The values of molar extinction coefficients were in the range 14,640–23,530 M^−1^cm^−1^. The PL spectra were measured at 290 nm excitation.

wavelength using polymer concentration of 10^−4^. Table 1 compiles the optical data of this polymer series and several interesting points are concluded:

**Table 1:** The optical properties of polyamides **36–45**

Polym. no	λ_abs_ (nm)	ε (M^−1^ cm^−1)^	λ_em_ (nm) (λ_ex_ at 290 nm)
**36**	282	22,730	346, 400, 580
**37**	277, 337 (sh)	21,940	346, 580
**38**	286, 337 (sh)	23,530	346, 580
**39**	279	21,370	346, 580
**40**	283	19,270	346, 580
**41**	280	19,320	346, 420, 580
**42**	280, 337 (sh)	22,240	346, 580
**43**	278, 337 (sh)	21,610	346, 580
**44**	280	14,640	346, 580
**45**	287	20,280	346, 580

- Relative to the unsubstituted polyamide, all substituted polymers showed slightly shifted absorption peaks due to the electronic effect of the substituent that increase the electron density, thereby leading to a relatively large energy band gap for π–π^*^ transitions.
- The fluorescence emission spectra of all polyamides exhibited two emission peaks at 346 nm and 580 nm.
- The orange emission observed for all polyamides at 580 nm could be attributed to the substituent’ electronic effect.
- Pyridine containing polyamide **41** exhibited slightly blue shifted absorption band relative to its phenylene analogue **36** while its emission showed a red shifted emission peak at 420 nm. Compared to a benzene ring, pyridine has a greater electron affinity and better electron-transporting properties.

##### The optical properties of the polyamides **46–51**

The optical properties of pyrimidine containing polymers **46**–**51** showed the values of molar extinction coefficients are in the range 43,600–74,100 M^−1^ cm^−1^ and the optical data are collected in Table 2. From the UV–vis spectral data given in Table 2 several remarks are found:

**Table 2:** Optical properties of polyamides **46–51**

Polym. no	λ_abs_ (nm)	ε (M^−1^ cm^−1)^	λ_em_ (nm) (λ_ex_ at 275 nm)
**46**	267	53,900	346, 413 (sh), 550
**47**	265, 312 (sh)	68,600	346, 433, 550
**48**	266, 331 (sh)	57,900	346, 410, 550
**49**	275	59,100	346, 454 (sh), 550
**50**	275, 328 (sh)	50,000	346, 450, 550
**51**	275	62,700	346, 428 (sh), 550

- Pyrimidine containing polyamide **46** exhibited a blue shifted absorption peak at 267 nm relative to its phenylene analogue **36**.
- Introduction of one methyl substituent led to a new absorption at 312 nm while the presence of two methyl substituents red-shifted the peak to 331 nm.
- Pyridine containing polyamides **49**–**51** exhibited similar absorption peaks at 275 nm and no further changes were noticed in presence of substitution. Relative to their phenylene analogues **46**–**48**, these series showed red shifted absorption (up to 10 nm).
- Green emission observed in this series at 550 nm in addition to the combined peak at 346 nm.

##### The optical properties of the polyamides **52–57**

The optical properties of pyrimidine containing polymers **52**–**57** showed the values of molar extinction coefficients are in the range 48,400–74,100 M^−1^ cm^−1^ and the optical data are collected in Table 3. From the UV–vis spectral data several remarks are found:

**Table 3:** Optical properties of polyamides **52–57**

Polym. no	λ_abs_ (nm)	ε (M^−1^ cm^−1)^	λ_em_ (nm) (λ_ex_ at 285 nm)
**52**	280	51,500	348, 432 (w), 482 (w), 572
**53**	278	67,500	348, 404 (sh), 572
**54**	283	74,100	348, 423, 482, 572
**55**	281	74,000	346, 433, 572
**56**	281	48,400	346, 572
**57**	283	43,600	346, 572

- All polymers in this series exhibited yellow emissions at 572 nm. No change upon phenylene/pyridine exchange except polymer **56** in which replacement red shifted the absorption. Furthermore, methyl substitution red-shifted the absorption bands.
- This series of polyamides exhibited red-shifted absorptions and emission peaks relative to their pyrimidine-containing polymers. This could be attributed either to the sulfonamide’s electronic effect or the increase of molecular polarizability which reduce the energy level separation.

#### Thermal analysis

##### Thermal properties of the polyamides **36**-**45**

The thermal properties of the prepared polymers were evaluated by differential thermo gravimetric (DTG) and differential thermal analysis (DTA) techniques. Thermal stability of the polymers was studied in the range 20–700 °C (char yield), Table 4. Structure–property relationship demonstrated an interesting connection between a single structure change and its thermal property.

**Table 4:** Thermoanalytical data of the polymers **36–45**

Polym. no	Steps	T(^°^C) range	% wt loss	Residue (%)	T_d_ (^°^C)
**36**	I	20–303	17.06	1.8	288
	II	303–438	16.25		404
	III	438–666	64.89		628
**37**	I	20–263	8.78	0.7	242
	II	263–463	43.6		373
	III	463–676	46.91		611
**38**	I	20–291	25.22	1.74	263
	II	291–435	27.52		380
	III	435–643	45.51		586
**39**	I	20–298	14.52	0	240
	II	298–466	20.9		389
	III	466–634	65.8		590
**40**	I	20–201	6.86	5.32	133
	II	201–412	13.94		361
	III	412–656	73.89		607
**41**	I	20–338	11.39	8.96	130
	II	338–489	30.33		476
	III	489–596	41.08		579
	IV	596–701	8.23		653
**42**	I	20–296	10.22	4.19	229
	II	296–456	35.56		391
	III	456–680	50.02		605
**43**	I	20–290	11.02	2.4	270
	II	287–425	28.73		399
	III	425–640	57.83		580
**44**	I	34–360	18.35	6.71	252
	II	364–503	30.5		472
	III	503–700	44.42		665
**45**	I	21–410	19.63	8.06	365
	II	412–564	39.47		536
	III	564–621	28.95		605
	IV	621–670	3.87		636

Phenylene-containing polymers **36**–**40** exhibited similar degradation behavior. DTA analysis revealed that the polyamide **36** exhibited an endothermic peak at 440 °C and an exothermic peak at 634 °C. The TGA exhibited degradation processes at 158 °C (8.6 % wt loss), 303 °C (8.5 % wt loss), 438 °C (16.3 % wt loss) and 666 °C (64.9 % wt loss) leaving 1.8 % as a mass residue. The DTA analysis of **37** exhibited a single exothermic peak at 607 °C, while its TGA analysis exhibited degradations at 107 °C (1.5 % wt loss), 197 °C (3.7 % wt loss), 263 °C (3.6 % wt loss), 463 °C (43.6 % wt loss) and 676 °C (46.9 % wt loss) leaving 0.7 % of as a residue. DTA analysis of the polyamide **38** exhibited an exothermic peak at 588 °C, while its TGA chart exhibited degradation processes at 196 °C (4.6 % wt loss), 291 °C (20.7 % wt loss), 435 °C (27.5 % wt loss) and 643 °C (45.5 % wt loss) leaving 1.7 % mass residue. DTA analysis of the polyamide **39** exhibited an exothermic peak at 590 °C and its TGA exhibited degradation processes at 205 °C (11.2 % wt loss), 298 °C (3.3 % wt loss), 466 °C (20.9 % wt loss) and 634 °C (65.8 % wt loss) leaving 0 % mass residue. DTA analysis of the polyamide **40** exhibited an exothermic peak at 607 °C. The TGA exhibited degradation processes at 201 °C (6.9 % wt loss), 412 °C (13.9 % wt loss) and 656 °C (73.9 % wt loss) leaving 5.3 % mass residue.

**Fig. 4:**
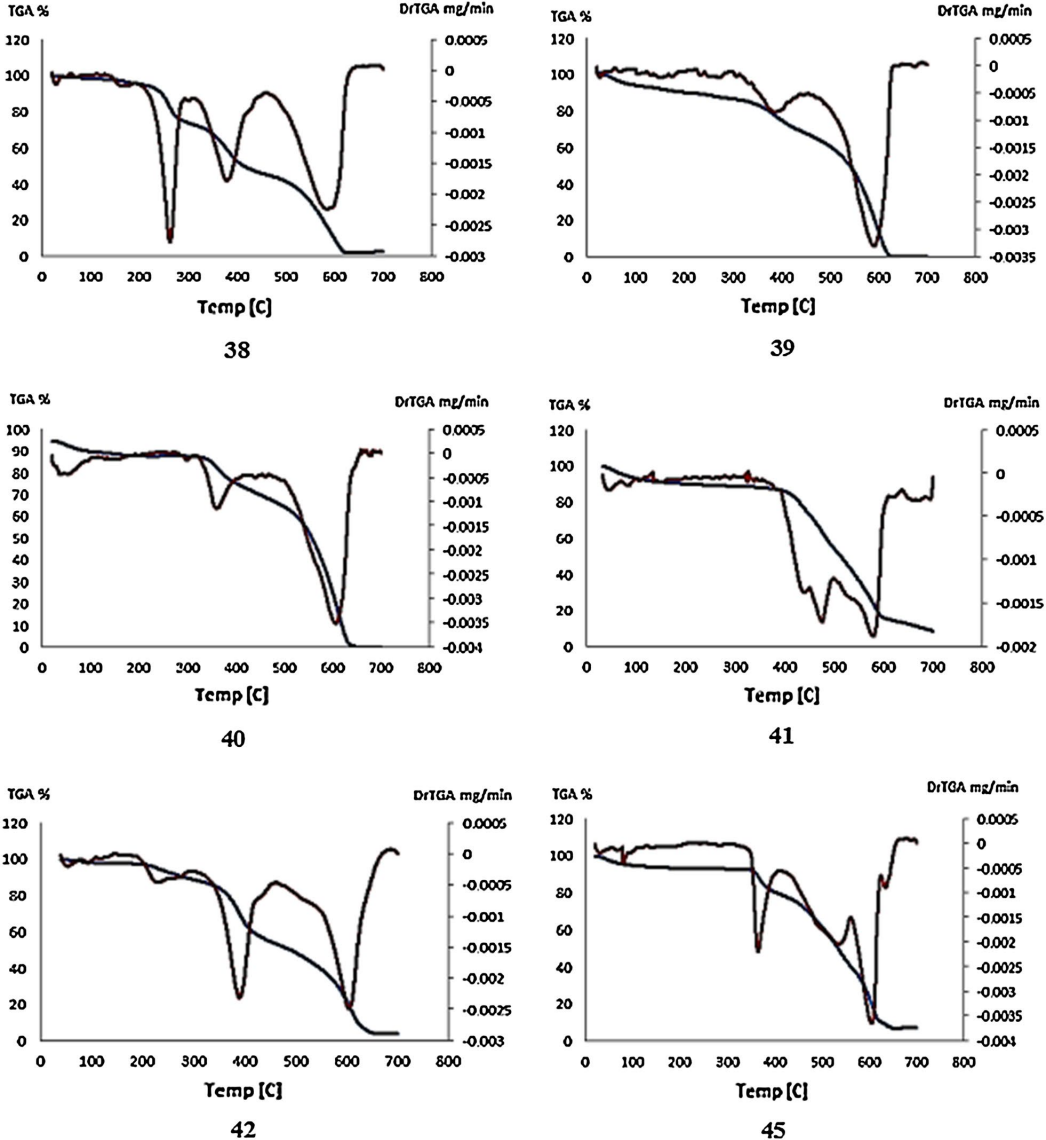
DTG curves of polymers **38–42** and **45**

Pyridine-containing polymers **41**–**45** exhibited slightly higher thermal stability compared to their phenylene analogues. The DTA of the polyamide **41** exhibited an endothermic decomposition peak at 437 °C and two other exothermic peaks at 480 °C and 581 °C, while its TGA analysis showed successive degradation processes at 338 °C (11.4 % wt loss), 489 °C (30.3 % wt loss), 596 °C (41.1 % wt loss) and 700 °C (8.2 % wt loss), leaving 8.96 % mass residue. The polyamide **42** exhibited an exothermic decomposition peak at 609 °C (DTA analysis), while TGA exhibited degradation processes at 146 °C (2.1 % wt loss), 296 °C (8.2 % wt loss), 456 °C (35.6 % wt loss) and 680 °C (50.0 % wt loss), leaving 4.19 % mass residue. The polyamide **43** exhibited two exothermic decomposition peaks at 405 °C and 577 °C (DTA analysis), while its TGA analysis exhibited degradation processes at 150 °C (3.4 % wt loss), 287 °C (7.7 % wt loss), 425 °C (28.7 % wt loss) and 640 °C (57.8 % wt loss), leaving 2.4 % as a residue. The polyamide **44** exhibited two exothermic decomposition peaks at 491 °C and 610 °C (DTA analysis), while the TGA analysis exhibited degradation processes atv153 °C (8.5 % wt loss), 364 °C (9.9 % wt loss), 503 °C (30.5 % wt loss), 640 °C (42.4 % wt loss) and 701 °C (2 % wt loss), leaving 6.7 % mass residue. Similarly, the polyamide **34** exhibited two exothermic decomposition peaks at 533 °C and 611 °C (DTA analysis), while the TGA exhibited degradation processes at 130 °C (5.8 % wt loss), 412 °C (13.8 % wt loss), 564 °C (39.5 % wt loss), 621 °C (28.9 % wt loss) and 670 °C (3.9 % wt loss), leaving 8.1 % as a residue. Thus, the introduction of pyridine moiety in the main chain of a polymer imparts thermal stability. The DTG curves of some representative examples of this series are shown in Fig. 4.

##### Thermal properties of the polyamides **46**–**51**

Pyrimidine-containing polymers **46**–**51** exhibited relatively high thermal stability compared to their analogues **36**–**45**, Table 5. The major degradation occurred in the range 400–700 °C leaving traces of the polymer as a mass residue. The introduction of the methyl substituent in the main chain of a polymer has no significant effect on the thermal stability. The DTA curve of the polyamide **46** exhibited an exothermic peak at 537 °C. The TGA curve showed successive degradation processes at 295 °C (14.2 % wt loss), 375 °C (10.3 % wt loss), 445 °C (13.4 % wt loss) and 699 °C (61.9 % wt loss), leaving 0.3 % of as a mass residue.

**Table 5:** Thermoanalytical data of the polymers **46–51**

Polym. no	Steps	T (°C)	% wt loss	Residue (%)	T_d_ (°C)
**46**	I	20–295	14.17	0.3	215
	II	295–375	10.26		370
	III	375–445	13.36		440
	IV	445–699	61.89		612
**47**	I	20–467	26.71	1.84	443
	II	467–550	19.41		527
	III	550–701	52.2		626
**48**	I	20–291	11.58	3.32	215
	II	291–403	16.64		354
	III	403–597	68.46		542
**49**	I	25–197	10.16	0.23	144
	II	197–398	24.1		362
	III	398–631	65.5		578
**50**	I	54–205	8.45	3.7	135
	II	205–379	15.17		349
	III	379–606	72.64		555
**51**	I	26–213	9.03	0	178
	II	213–275	11.67		257
	III	275–424	17.28		351
	IV	424–670	62.12		633

The polyamide **47** exhibited an endothermic peak at 592 °C and another exothermic peak at 656 °C (DTA analysis). The TGA showed degradation processes at 443 °C (26.71 % wt loss), 550 °C (19.4 % wt loss) and 700 °C (52.2 % wt loss), leaving 1.8 % mass residue. The polyamide **48** exhibited an exothermic peak at 607 °C (DTA analysis). The TGA analysis showed successive degradation processes at 215 °C (11.58 % wt loss), 403 °C (16.6 % wt loss) and 597 °C (68.5 % wt loss), leaving 3.32 % as a residue.

Pyridine-containing polyamide **49** exhibited an exothermic decomposition peak at 633 °C (DTA analysis). The TGA analysis exhibited four degradation processes at 99 °C (6.9 % wt loss), 197 °C (3.3 % wt loss), 398 °C (24.1 % wt loss) and 631 °C (65.5 % wt loss), leaving 0.23 % as a mass residue. The DTA analysis of the polyamide **50** exhibited an exothermic peak at 555 °C. The TGA analysis showed three degradation processes at 205 °C (8.5 % wt loss), 379 °C (15.2 % wt loss) and 606 °C (72.6 % wt loss), leaving 3.7 % as a residue. The polyamide **51** exhibited an exothermic peak at 574 °C (DTA analysis). The TGA analysis showed five degradation processes at 137 °C (7.0 % wt loss), 213 °C (2 % wt loss), 275 °C (11.7 % wt loss), 424 °C (17.3 % wt loss) and 670 °C (62.1 % wt loss), leaving 0 % residue.

##### Thermal properties of the polyamides **52**–**57**

Thermal properties of the polyamides **52–57** are collected in Table 6 and the results revealed comparable thermal stability. As indicated by the DTA chart, the polyamide **52** exhibited two exothermic decomposition peaks at 491 °C and 539 °C. The DTG curve exhibited degradation processes at 174 °C (8.1 % wt loss), 385 °C (34.9 % wt loss), 451 °C (11.2 % wt loss) and 622 °C (45.5 % wt loss), leaving 0.24 % as a residue. The polyamide **53** showed two exothermic peaks at 533 °C and 596 °C (DTA analysis). The TGA exhibited degradation processes at 73 °C (5.4 % wt loss), 497 °C (39.7 % wt loss) and 663 °C (53.3 % wt loss), leaving 1.67 % as a mass residue.

**Table 6:** Thermoanalytical data of the polymers **52**–**57**

Polym. no	Steps	T (°C)	% wt loss	Residue (%)	T_d_ (°C)
**52**	I	20–385	43.05	0.24	307
	II	385–451	11.2		440
	III	451–622	45.49		591
**53**	I	20–497	45.05	1.67	493
	II	497–663	53.29		596
**54**	I	20–340	28.19	3.05	315
	II	340–450	21.4		440
	III	450–502	19.11		487
	IV	502–590	28.26		540
**55**	I	20–303	13.51	1.26	160
	II	303–411	19.27		371
	III	411–647	65.96		592
**56**	I	20–392	24.03	3.18	376
	II	392–566	53.3		560
	III	566–629	19.5		587
**57**	I	20–410	23.93	10.1	386
	II	410–528	21.0		492
	III	528–600	12.9		559
	IV	600–700	32.98		656

**Fig. 5:**
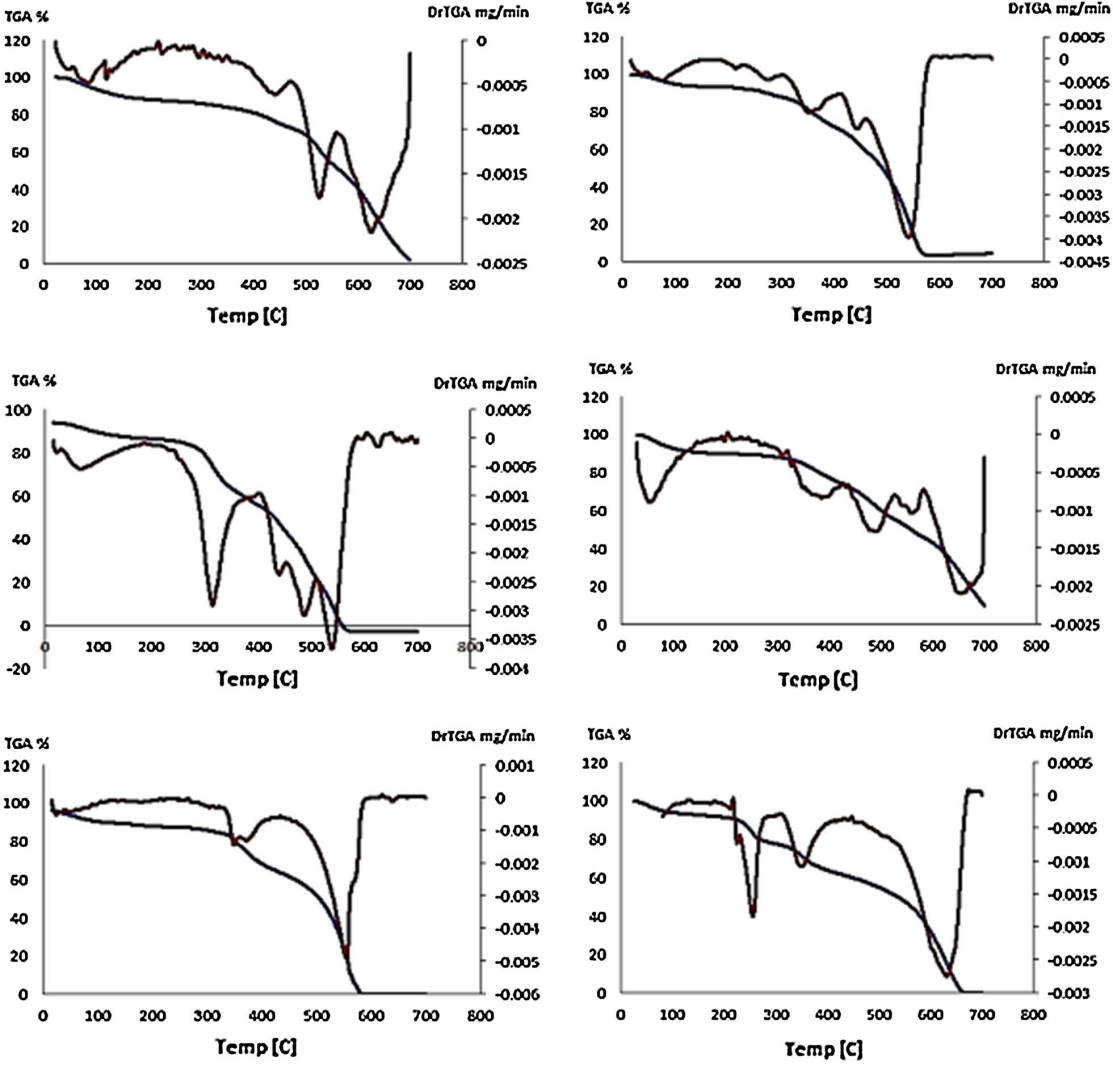
DTG curves of polymers **47**, **48**, **51**, **53**, **54** and **57**

The polyamide **54** exhibited two exothermic peaks at 440 and 581 °C (DTA analysis). The TGA analysis showed five degradation processes at 133 °C (6.1 % wt loss), 340 °C (22.1 % wt loss), 450 °C (21.4 % wt loss), 502 °C (19.1 % wt loss) and 590 °C (28.3 % wt loss), leaving 3.1 % mass residue. The DTA analysis revealed that **55** exhibited two endothermic peak at 460 °C and 584 °C, while the TGA analysis exhibited degradation processes at 194 °C (10.0 % wt loss), 303 °C (3.5 % wt loss), 411 °C (19.3 % wt loss) and 647 °C (65.9 % wt loss), leaving 1.3 % of the polymer as a mass residue. The polyamide **56** exhibited two exothermic decomposition peaks at 563 and 589 °C (DTA), while the TGA chart showed degradation processes at 160 °C (7.9 % wt loss), 392 °C (16.0 % wt loss), 566 °C (53.3 % wt loss) and 629 °C (19.5 % wt loss), leaving 3.2 % as a remaining mass residue. The DTA data of the polyamide **57** showed an endothermic peak at 372 °C and exothermic peak at 593 °C. The TGA analysis exhibited degradation processes at 81 °C (5.6 % wt loss), 410 °C (18.2 % wt loss), 528 °C (21.0 % wt loss), 600 °C (12.9 % wt loss) and 700 °C (32.9 % wt loss), leaving 10.1 % as a mass residue. The DTG curves of some representative examples of this series are shown in Fig. 5.

In summary, pyrimidine-containing polyamides exhibited relatively higher thermal stability compared to their sulfonamido-pyrimidine analogues. This may be explained by the feature of the supramolecular structure, namely by a high density of packing of polymeric chains, realized through a level-by-level stacking of these chains. With such stacking a strong intermolecular interaction between the amide fragments of adjacent polymeric chains is provided. However, in the case of the former polymers, the interchain interaction can occur due to specific contacts between the amide fragments of one chain and the nitrogen atoms of the pyrimidine cycles of the other chain. Owing to this fact the pyrimidine cycles serve as an additional amplifier of the interchain interaction in polyamides, thus causing the strength and thermal stability to increase. The presence of a sulfonamide group adjacent to pyrimidine, as in the case of the latter polymer series, led to decrease thermal stability. This may be attributed to the acidic nature of the hydrolyzable group that retain the high polarity and thus, the polymer degraded before melting stage. Nevertheless, the methyl substitution enhanced the thermal stability in this series.

##### Calculations of limiting oxygen index

Flammability of polymers is one important property which could limit their applications [30]. Despite the fact that high-performance polymeric materials offer many advantages over conventional metals, their flammability and possible release of toxic byproducts increase the fire risk and thus the introduction of flame-retardant additives are the easiest way to diminish the polymer flammability. The flame retardancy is evaluated by limiting oxygen index (LOI). The LOI is defined as the minimum oxygen concentration needed in an inert gas medium for the material to achieve burning after ignition. The LOI is a measure of the ratio of oxygen to other gases in the air surrounding a substrate. A material with an LOI of greater than 21 % but less than 28 % would be considered “slow burning” while a material with an LOI of greater than 28 % would be considered “self-extinguishing”. Char yield can be used as criteria for evaluating LOI of the polymers in accordance with Van Krevelen and Hoftyzer equation [31]; LOI = 17 + 0.4CR, where CR = chars yield. The calculated LOI values of all polymers based on their char yield were less than 28, Tables 7,8.

**Table 7:** The char yields and LOI values of the polyamides **36**–**45**

No	**36**	**37**	**38**	**39**	**40**	**41**	**42**	**43**	**44**	**45**
R*	1.8	0.7	1.7	0.0	5.3	8.96	4.19	2.4	6.71	8.06
LOI	17.7	17.3	17.6	17	19.1	20.6	18.7	18.0	19.7	20.2

R* Char residue

**Table 8:** The char yields and LOI values of the polyamides **46**–**57**

No	**46**	**47**	**48**	**49**	**50**	**51**	**52**	**53**	**54**	**55**	**56**	**57**
R*	0.3	1.84	0.3	0.23	3.7	0.0	0.24	1.67	3.05	1.26	3.18	10.1
LOI	17.1	17.7	17.1	17.1	18.5	17.0	17.1	17.6	18.2	17.5	18.3	21.0

R* Char residue

##### Calculations of thermodynamic parameters

The thermodynamic parameters of decomposition processes of polymers, namely, activation energy *∆E* enthalpy (∆*H*), entropy (∆*S*) were evaluated by employing the Horowitz-Metzger equation [32], Additional file 1: Tables S1, S2. The order of chemical reactions (n) was calculated via the peak symmetry method by Kissinger [33]. The asymmetry of the peak, *S*, is calculated as follows:

1 $${\text{S}} = 0. 6 3 {\text{n}}^{ 2}$$

2 $${\text{n}} = 1. 2 6 { }\left( {{\text{a}}/{\text{b}}} \right)^{ 1/ 2}$$

The value of the decomposed substance fraction, αm, at the moment of maximum development of reaction (with T = T_m_) being determined from the relation (3):

3 $$\left( { 1- \alpha_{\text{m}} } \right) = {\text{n}}^{{ 1/ 1- {\text{n}}}}$$

The values of collision factor, Z, can be obtained in case of Horowitz Metzger by making the use of the relation (4):

4 $$z = \frac{E}{RT_{m}}\phi \exp \left( {\frac{E}{{RT2_{m} }}} \right) = \frac{{KT_{m} }}{h}\exp \left( {\frac{\varDelta S^*}{R}} \right)$$

where *S* is the entropies of activation, R represents molar gas constant, Φ rate of heating (K s^−1^), K the Boltzmann constant, and h the Planck’s constant [34]. The change in enthalpy (∆H) for any phase transformation taking place at any peak temperature, Tm, can be given by the following equation: ∆ *S* = ∆*H*/Tm. Based on least square calculations, the Ln ∆T versus 1,000/T plots for all complexes, for each DTA curve, gave straight lines from which the activation energies were calculated according to the reported methods [35]. The slope is of Arrhenius type and equals −E/R.

The kinetic data obtained from the nonisothermal decomposition of the prepared polyamides series containing chloroaromatic pendent moiety **36–45** are given in Additional file 1: Table S1. The following trends and conclusions may be achieved:

1. The calculated values of the collision number, *Z*, showed a direct relation to *E*a. The maximum and minimum *Z* values for polyamides **36**–**40** derived from isophthaloyl dichloride were 7.28 S^−1^ and 1.014 S^−1^ and that derived from pyridine 2,6-dicarbonyl dichloride **41**–**45** were 12.69 S^−1^ and 1.02 S^−1^ suggesting different degradation mechanisms with variable speeds. The values of the decomposed substance fraction, α_m_ for the polyamides **36**–**40** at the maximum development of the reaction are of nearly the same magnitude and lie within the range 0.48–0.64.
2. The change of entropy values, ∆*S,* for all polymers has nearly the same magnitude lie within the range −0.23 to −0.25 kJ K^−1^ mol^−1^ and the negative signs of the entropy suggest ordered transition states, i.e., in a less random molecular configuration. The fractions appeared in the calculated order of the thermal reactions, n, confirmed that the reactions proceeded in complicated mechanisms.
3. Activation energies (∆E) of polyamides **36**–**40** demonstrated lower values compared to their partners **41–45**. The first and second decomposition steps in some polymers have nearly equal ∆E values, indicating similar degradation mechanism.
4. The enthalpy (∆H) of polyamides **36–40** demonstrated higher values compared to their partners **41**–**45**, respectively, and the negative values demonstrated the exothermic decomposition processes.

The kinetic data obtained from the nonisothermal decomposition of the polymers **46**–**57** are given in Additional file 1: Table S2. The following trends and conclusions may be achieved:

1. The maximum and minimum collision number *Z* values for polyamides **46**–**50** are 3.45 and 0.93 S^−1^, respectively, while that for the polyamides **51**–**57** are 26.6 and 1.03 S^−1^ suggesting different degradation mechanisms with variable speeds. Noteworthy, the collision number Z values for polyamides containing sulfonamide group are 1.57, 1.2, 0.9, respectively, suggesting similar degradation mechanisms. The values of the decomposed substance fraction, α_m_ for polyamides at the maximum development of the reaction are of nearly the same magnitude and lie within the range 0.33–0.73.
2. The entropy values, ∆*S,* for all polymers have nearly the same magnitude and were in the range −0.22 to −0.25 kJ K^−1^ mol^−1^. The observed negative signs clearly demonstrated that the transition states are more ordered, i.e., in a less random molecular configuration. The fractions appeared in the calculated order of the thermal reactions, n, confirmed that the reactions proceeded in complicated mechanisms.
3. Activation energies (∆E) of polyamides **46–50** demonstrated lower values compared to their partners **51–57**. Noteworthy, polyamides containing sulfonamide exhibited higher ∆E and methyl substitution produced polymer have high ∆E than their unsubstituted analogs.
4. The enthalpy (∆H) of polyamides **46–50** demonstrated higher values compared to their partners **51**–**57** and the negative values demonstrated exothermic decomposition processes.

### Biological properties

#### Antimicrobial activity

The antimicrobial activity of the polyamides series **36**–**57** were examined against a variety of microorganisms included fungi such as: *A. fumigatus* RCMB 02568, *S. racemsum* RCMB 05922, *G. candidum* RCMB 05097 and *C. albicans* RCMB 05036; gram positive bacteria such as: *S. pneumoniae* RCMB 010010 and *B. subtilis* RCMB 010067; and gram negative bacteria such as: *P. aeruginosa* RCMB 010043 and *E. coli* RCMB 010052. In all cases, the diffusion agar technique was applied and the antimicrobial activity results are collected in Additional file 1: Tables S3–S5.

##### Antimicrobial activity of polymeric series **36**–**45**

Chloro aromatic compounds played a vital role in the development of different medicinal agents where chlorine is electronegative, and therefore oxidizes peptide link and denatures proteins. Exposure of strains of *E. coli, Pseudomonas* spp. and *Staphylococcus* spp. to lethal doses causes a decrease in ATP production. Chlorine acts on the permeability of the external membrane of *E. coli* through a primary lethal phenomenon which consists in a substantial leakage of K + ions; such leakage does not occur for macromolecules. Sub-lethal doses inhibit cellular respiration due to a nonspecific oxidizing effect (bactericidal effect) [36].

Results of antimicrobial activity of polyamides **36–40**, derived from isophthaloyl chloride, and the comparative activity of currently used antibacterial and antifungal agents are presented in Additional file 1: Table S3. Thus, the introduction of choro substituents clearly enhanced the antimicrobial activity against all fungi and *B. subtilis* with inhibition zone diameters ranging between 13.4 and 19.6 mm. Compared to other analogues, the polyamide containing *p*-chloro substituent showed higher activity against the tested microorganisms.

The antimicrobial activity comparative tests of the polymeric series **41–45**, derived from pyridine 2,6-dicarbonyl dichloride, are presented in Additional file 1; Table S4. Polyamides containing both sulfonamide and chloro substituents showed higher antimicrobial activity against all fungi and gram positive bacteria. The presence of such bioactive groups in the backbone of the polymer play the key role in catalyzing both biological and chemical systems. Compared to their analogues **25**–**29**, the polyamides **30**–**34** showed relatively higher antibacterial activities against all tested microorganisms.

##### Antimicrobial activity of polymeric series **46**–**57**

Sulfonamides are chemotherapeutic agents which display various biological interactions, including inhibition of carbonic anhydrase and affecting insulin releasing in addition to their antimicrobial, antitumor and anti-inflammatory activities. The antimicrobial activity of the amido- and sulfonamido-pyrimidine containing polymers **46–57** are presented in Additional file 1: Tables S4, S5. From the screening results, the following remarks are concluded:

- Pyrimidine-containing polyamides exhibited high antifungal activity than their analogues containing sulfonamidopyrimidine pendant structures. Thus, the presence of sulfonamide structures in such polymeric series considerably alters the antimicrobial activity of the polymer. The polyamides **46**–**48** exhibited remarkable antifungal activities against *A. fumigatus* and, interestingly, the observed activity were more potent than those of the reference *AmphotericinB*. Over 80 % of the reported *Aspergillus*-related cases, such as extrinsic allergic alveolitis, asthma, allergic sinusitis, chronic eosinophilic pneumonia, hypersensitivity pneumonitis, and allergic bronchopulmonary aspergillosis are most frequently caused by *A. fumigatus* [37].

Moreover, introduction of methyl substituents in case of the polyamides **47** and **48** produced potent antifungal polymers against *S. racemosum* and, the noteworthy, the activity were higher than the reference *AmphotericinB* and thus, the introduction of a methyl group to the pyrimidine promotes antifungal activity. *S. Racemosum* is well known to cause skin and soft tissue infection and fungal rhinosinusitis [38].

Sulfonamidopyrimidine-containing polyamides analogues **51–53** exhibited higher antibacterial activity against gram negative bacteria than their analogues **46**–**48**. Thus, replacement of the amide linkage by sulfonamide linkage promoted specifically the antibacterial activity against gram negative type. Noteworthy, relative to the reference antibiotic *Gentamicin*, the polyamide **54** exhibited comparable antibacterial activity against gram negative bacteria. It has been reported that *P. aeruginosa* is the most common pathogen causing chronic infection in people with cystic fibrosis (an inherited disease that affects the lungs, digestive system and sweat glands) [39].

- Pyrimidine-containing polymer analogue **52** exhibited remarkable antibacterial activities against *S. pneumoniae*, a gram positive bacterium and *P.aeruginosa* a gram negative bacterium. In both cases, activities were more potent compared to the references antibiotics used. Thus, the polyamide **52** is likely to be a promising broad spectrum antibacterial agent.
- In general, polymers have pyrimidinoamide linkages exhibited lower activities toward gram positive bacteria than their analogues have sulfonamidopyrimidine linkage.
- The polyamide analogue **57** exhibited promising antibacterial activities against both gram positive and gram negative bacteria. Interestingly, its activity as reflected by the inhibition zone diameter is higher than the reference antibiotic *Gentamicin*.

#### Antimicrobial activities’ statistical analyses

**Fig. 6:**
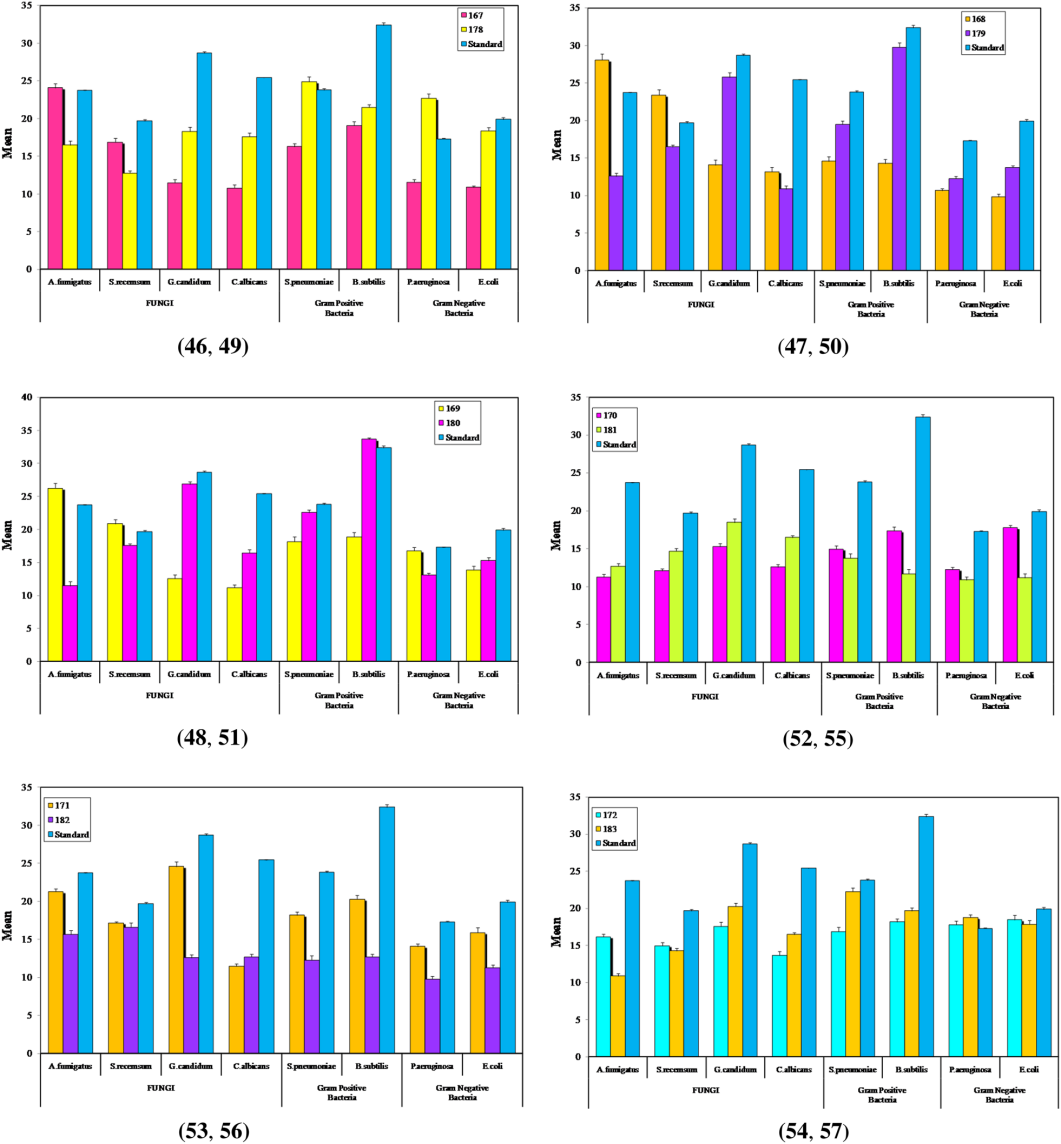
Inhibition zone values of the potent polymers **46–55**, **57** and the standard against fungi: *A. fumigatus*, *S. racemsum*, *G. candidum* and *C. albicans*; and bacteria; gram positive bacteria: *S. pneumoniae* and *B. subtilis*; and gram negative bacteria: *P. aeruginosa* and *E. coli*.

The antimicrobial activity data of the most promising polymers were analyzed against their corresponding controls using SPSS software package version 18.0 (SPSS, Chicago, IL, USA). These included the cases at which the structures **35**–**37**, **38**, **40**, **43** and **46** exceeded the activities of the examined reference antimicrobial agents. Quantitative data were analyzed using an F-test and the results are presented in Additional file 1: Table S7 and Fig. 6. The p value was assumed to be significant at ≤0.05. From the screening results, the following remarks are concluded:

- *A. fumigatus* was significantly (p < 0.001) sensitive to the tested pyrimidine-containing polymers **46**–**48** compared to the control.
- *S. Racemosum* was significantly (p < 0.003) sensitive to substituted pyrimidine-containing polymers **47**–**48** compared to the control.
- *B. subtilis* was significantly (p < 0.004) sensitive to the polymer **50** compared to the analogues **49** and **51** and the control.
- *S. pneumonia* was significantly (p < 0.001) sensitive to the polymer **52** which exhibited broad antibiotic spectrum compared to the analogues **53**–**54** and the control.

Although these polymers have shown remarkable antimicrobial activity, further studies need to be conducted to ascertain the exact mechanism of the activity and the minimal inhibitory concentration.

## Conclusions

A series of aromatic polyamides containing substituted halogenated aromatic, pyrimidineamido- and pyrimidinesulfonamido pendent structures in bulk and nanoscale were synthesized and screened for their antimicrobial activity against microorganisms. The SEM analysis of polyamides indicated that most of them were organized as well defined nano sized spheres but in case of pyridine and pyrimidine containing polyamides small amount of aggregated nanospheres were also observed. Thermal analysis of the polymers was studied in the temperature range 20–700 °C and results showed comparable thermal behavior.

The optical results showed that polyamides series containing chloroaromatic pendent moiety exhibited orange emission at 580 nm. The pyrimidineamido polymeric series showed green emission 550 nm while their pyrimidinesulfonamido analogues exhibited yellow emission 572 nm in addition to the blue emission at 482 nm. Interestingly, structural modification via benzene/pyridine interchange resulted in red shifted emission peaks in most cases and this could be attributed to the localized lone pair of electrons in the sp^2^ orbital of the nitrogen atom which offer the polyamide a greater electron affinity and better electron-transporting properties.

Biological results showed that the halogenated polyamides exhibited good antimicrobial activity against most tested microorganisms. The amido- and sulfonamidopyrimidine containing polymers exhibited most potent antimicrobial agents in the present series. Polymers having pyrimidinoamide linkages exhibited lower activities toward gram positive bacteria than their analogues have sulfonamidopyrimidine linkage. Relative to the reference antibiotic *Gentamicin*, the polyamide **54** exhibited comparable antibacterial activity against gram negative bacteria (*P. aeruginosa*); the most common pathogen causing chronic infection in people with cystic fibrosis. Pyrimidine-containing polymer analogues **52** and **57** exhibited remarkable antibacterial activities against gram positive and gram negative bacteria. In both cases, activities were more potent compared to the references antibiotics used. Thus, these polyamides are likely to be promising broad spectrum antibacterial agents and deserve further investigation in order to clarify the mode of action at the molecular level.

## Additional file

Additional file 1:
**Table S1.** Kinetic parameters of the polymers **36**–**45**. **Table S2.** Kinetic parameters of the polymers **46**–**57**. **Table S3.** Antimicrobial activity of polyamides **25**–**29** (discs Ø 6 mm). **Table S4.** Antimicrobial activity of polyamides **41**–**45** (discs Ø 6 mm). **Table S5.** Antimicrobial activity of polyamides **46**–**51** (discs Ø 6 mm). **Table S6.** Antimicrobial activity of polyamides **52**–**57** (discs Ø 6 mm). **Table S7.** Statistical analysis of some polyamides exhibited promising antimicrobial agents.
