# Supplementary material for: Synthesis and biological evaluation of new nanosized aromatic polyamides containing amido- and sulfonamidopyrimidines pendant structures
Source: Chem Cent J. 2015 Aug 19;9:44. doi: 10.1186/s13065-015-0123-2 (PMC4540749; doi:10.1186/s13065-015-0123-2)
Supplement: Additional file 1: — Table S1. Kinetic parameters of the polymers 36–45. Table S2. Kinetic parameters of the polymers 46–57. Table S3. Antimicrobial activity of polyamides 25–29 (discs Ø 6 mm). Table S4. Antimicrobial activity of polyamides 41–45 (discs Ø 6 mm). Table S5. Antimicrobial activity of polyamides 46–51 (discs Ø 6 mm). Table S6. Antimicrobial activity of polyamides 52–57 (discs Ø 6 mm). Table S7. Statistical analysis of some polyamides exhibited promising antimicrobial agents. [file 13065_2015_123_MOESM1_ESM.docx]

**Table S1:** Kinetic parameters of the polymers **36**-**45**

| **No** | **Peak**  **Type** | **Slope**  **(DTA)** | **∆E** | **A** | **B** | **S** | **N** | **α_m_** | **T_m_ (K)** | **Z**  **(s^-1^)** | **∆S** | **∆H** | **R^2^** |
| --- | --- | --- | --- | --- | --- | --- | --- | --- | --- | --- | --- | --- | --- |
| **36** | exo  exo | -32.57  -16.70 | 270.79  138.88 | 2.0  4.6 | 2.0  1.5 | 1.0  3.07 | 1.26  2.21 | 0.59  0.48 | 713  907 | 7.28  2.29 | -0.23  -0.24 | -102.05  -154.9 | 0.90  0.99 |
| **37** | exo | -13.25 | 110.18 | 4.2 | 2.5 | 2.65 | 1.63 | 0.54 | 880 | 1.89 | -0.24 | -149.21 | 0.99 |
| **38** | exo | -17.39 | 144.58 | 4.0 | 2.0 | 2.01 | 1.79 | 0.52 | 861 | 2.59 | -0.24 | -142.83 | 0.97 |
| **39** | exo | -24.01 | 199.59 | 3.4 | 1.4 | 2.4 | 1.96 | 0.50 | 863 | 2.178 | -0.24 | -144.12 | 0.98 |
| **40** | exo | -11.93 | 99.22 | 3.8 | 1.5 | 2.54 | 2.01 | 0.50 | 880 | 1.014 | -0.25 | -152.32 | 0.90 |
| **41** | endo  exo  exo | -36.67  -19.44  -43.91 | 304.91  161.59  365.07 | 2.0  0.5  2.5 | 2.0  1.0  1.5 | 1.0  0.5  1.61 | 1.26  0.89  1.6 | 0.59  0.65  0.54 | 710  753  854 | 5.07  2.2  4.3 | -0.23  -0.24  -0.24 | -102.62  -116.4  -138.63 | 0.93  0.89  0.99 |
| **42** | exo | -29.61 | 246.2 | 3.0 | 2.2 | 1.36 | 1.47 | 0.56 | 882 | 2.63 | -0.24 | -148.07 | 0.99 |
| **43** | exo  exo | -13.68  -11.41 | 113.77  94.85 | 1.0  4.0 | 0.5  1.5 | 1.99  1.2 | 1.78  1.38 | 0.52  0.49 | 678  850 | 1.83  1.02 | -0.24  -0.25 | -98.35  -144.5 | 0.91  0.99 |
| **44** | exo  exo | -58.68  -44.78 | 487.85  372.28 | 4.8  2.5 | 0.5  1.5 | 0.56  1.67 | 0.94  1.63 | 0.64  0.54 | 764  883 | 12.69  6.89 | -0.23  -0.23 | -111.95  -143.42 | 0.97  0.96 |
| **45** | exo  exo | -24.23  -37.98 | 201.45  315.82 | 1.0  1.8 | 0.5  0.5 | 1.99  3.6 | 1.78  2.39 | 0.52  0.46 | 806  884 | 4.12  5.73 | -0.24  -0.23 | -126.94  -144.59 | 0.97  0.99 |

ΔE: activation energy, S: asymmetry of the peak, n: order of chemical reaction, αm: decomposed substance fraction at the moment of maximum development of reaction with (T=T_m_), Z: collision factor (s^-1^), ∆S: entropy in KJ/mol, ∆H: enthalpy in KJ/mol, R^2^: coefficient of determination.

**Table S2:** Kinetic parameters of the polymers **46**-**57**

| **No** | **Peak**  **Type** | **Slope**  **(DTA)** | **∆E** | **A** | **B** | **S** | **N** | **α_m_** | **T_m_ (K)** | **Z**  **(s^-1^)** | **∆S** | **∆H** | **R^2^** |
| --- | --- | --- | --- | --- | --- | --- | --- | --- | --- | --- | --- | --- | --- |
| **46** | exo | -12.93 | 107.5 | 3.8 | 1.5 | 2.5 | 2.00 | 0.50 | 810 | 2.09 | -0.24 | -130.84 | 0.99 |
| **47** | endo  exo | -36.84  -23.22 | 306.29  193.07 | 1.0  1.8 | 1.3  2.2 | 0.77  0.82 | 1.10  1.14 | 0.61  0.61 | 865  929 | 3.45  3.11 | -0.24  -0.24 | -142.37  -158.94 | 0.91  0.98 |
| **48** | exo | -11.46 | 95.27 | 4.5 | 3.0 | 1.5 | 1.54 | 0.55 | 880 | 1.62 | -0.25 | -150.05 | 0.98 |
| **49** | exo | -19.02 | 158.12 | 3.8 | 1.5 | 2.5 | 2.01 | 0.50 | 906 | 1.57 | -0.25 | -156.63 | 0.96 |
| **50** | exo | -12.97 | 107.82 | 4.5 | 1.3 | 3.46 | 2.34 | 0.47 | 828 | 1.21 | -0.25 | -138.03 | 0.99 |
| **51** | exo | -10.38 | 86.32 | 3.8 | 1.8 | 2.11 | 1.83 | 0.52 | 847 | 0.93 | -0.25 | -144.19 | 0.98 |
| **52** | exo  exo | -12.75  -58.33 | 106.04  484.95 | 2.1  2.1 | 1.2  3.0 | 1.75  0.7 | 1.67  1.05 | 0.53  0.62 | 764  812 | 1.36  6.60 | -0.25  -0.23 | -121.03  -126.30 | 0.98  0.99 |
| **53** | exo  exo | -8.839  -48.55 | 73.49  403.64 | 2.3  2.0 | 0.6  2.7 | 3.84  0.73 | 2.47  1.08 | 0.46  0.62 | 806  869 | 1.42  7.77 | -0.25  -0.23 | -131.77  -139.42 | 0.95  0.97 |
| **54** | exo  exo | -15.82  -31.35 | 131.52  260.64 | 0.7  1.9 | 0.5  2.8 | 1.4  0.68 | 1.49  1.04 | 0.56  0.63 | 713  854 | 3.25  4.93 | -0.24  -0.24 | -104.93  -137.88 | 0.99  0.99 |
| **55** | endo  endo | -62.08  -182.4 | 516.16  1516.3 | 0.5  0.5 | 2.5  0.9 | 0.2  0.56 | 0.56  0.94 | 0.73  0.64 | 733  857 | 9.03  26.6 | -0.23  -0.22 | -106.07  -130.46 | 0.99  0.99 |
| **56** | exo  exo | -14.74  -116.9 | 122.56  972.49 | 4.5  2.1 | 0.3  1.6 | 1.5  1.3 | 4.88  1.44 | 0.33  0.56 | 837  862 | 1.37  13.87 | -0.25  -0.23 | -139.52  -134.90 | 0.98  0.96 |
| **57** | endo  exo | -41.69  -11.81 | 346.62  98.19 | 0.5  3.5 | 1.0  2.8 | 0.5  1.25 | 0.89  1.41 | 0.65  0.57 | 645  866 | 7.55  1.03 | -0.23  -0.25 | -85.73  -148.52 | 0.81  0.99 |

ΔE: activation energy, S: asymmetry of the peak, n: order of chemical reaction, αm: decomposed substance fraction at the moment of maximum development of reaction with (T=T_m_), Z: collision factor (s^-1^), ∆S: entropy in KJ/mol, ∆H: enthalpy in KJ/mol, R^2^: coefficient of determination.

**Table S3:** Antimicrobial activity of polyamides **25-29** (discs Ø 6mm).

| Microorganism | **36** | **37** | **38** | **39** | **40** | Standard |
| --- | --- | --- | --- | --- | --- | --- |
| **FUNGI** |  |  |  |  |  | *Amphotericin B* |
| *A. fumigatus* | 12.7±0.5 | 16.8±0.39 | 15.7±0.33 | **20.6±0.58** | 18.7±0.36 | 23.7±0.1 |
| *S. racemsum* | 10.8±0.4 | 13.4±0.58 | 13.8±0.25 | 16.7±0.33 | **16.9±0.27** | 19.7±0.2 |
| *G. candidum* | 15.1±0.4 | 19.6±0.19 | 18.3±0.34 | **22.4±0.36** | 13.4±0.65 | 28.7±0.2 |
| *C. albicans* | 14.7±0.4 | 15.9±0.44 | 15.2±0.5 | **17.6±0.58** | 12.8±0.4 | 25.4±0.1 |
| **(+) Gram Bacteria** |  |  |  |  |  | *Ampicillin* |
| *S. pneumoniae* | 17.3±0.4 | 16.7±0.36 | 16.9±0.58 | **18.3±0.25** | 12.9±0.63 | 23.8±0.2 |
| *B. subtilis* | 13.3±0.3 | 19.2±0.27 | 18.2±0.44 | **22.6±0.44** | 13.2±0.58 | 32.4±0.3 |
| **(-) Negative Bacteria** |  |  |  |  |  | *Gentamicin* |
| *P. aeruginosa* | 15.3±0.4 | 10.9±0.3 | 14.6±0.5 | **16.7±0.45** | 16.1±0.37 | 17.3±0.1 |
| *E. coli* | **18.3±0.5** | 13.6±0.5 | 11.9±0.63 | 17.8±0.44 | 12.8±0.44 | 19.9±0.3 |

**Table S4:** Antimicrobial activity of polyamides **41-45** (discs Ø 6mm).

| Microorganism | **41** | **42** | **43** | **44** | **45** | standard |
| --- | --- | --- | --- | --- | --- | --- |
| **FUNGI** |  |  |  |  |  | *Amphotericin B* |
| *A. fumigatus* | 15.3±0.55 | 17.6±0.58 | 13.6±0.25 | NA | **20.2±0.55** | 23.7±0.1 |
| *S. racemsum* | 13.4±0.35 | 15.4±0.25 | 14.9±0.34 | NA | 16.3±0.25 | 19.7±0.2 |
| *G. candidum* | 11.5±0.58 | 12.6±0.38 | 16.5±0.58 | NA | 22.4±0.58 | 28.7±0.2 |
| *C. albicans* | 13.1±0.3 | 10.9±0.4 | 11.7±0.34 | 9.7±0.3 | 19.6±0.33 | 25.4±0.1 |
| **(+) Gram Bacteria** |  |  |  |  |  | *Ampicillin* |
| *S. pneumoniae* | 17.5±0.44 | 12.3±0.58 | 14.6±0.58 | 15.7±0.6 | 18.9±0.44 | 23.8±0.2 |
| *B. subtilis* | 19.8±0.63 | 12.7±0.37 | 14.3±0.58 | 13.2±0.4 | 21.7±0.25 | 32.4±0.3 |
| **(-) Negative Bacteria** |  |  |  |  |  | *Gentamicin* |
| *P. aeruginosa* | 15.1±0.45 | 11.2±0.25 | 12.3±0.42 | 11.2±0.4 | 12.6±0.19 | 17.3±0.1 |
| *E. coli* | **18.9±0.25** | 9.5±0.37 | 9.4±0.44 | 15.7±0.5 | 15.4±0.44 | 19.9±0.3 |

**Table S5:** Antimicrobial activity of polyamides **46**-**51** (discs Ø 6mm).

| Microorganism | **46** | **47** | **48** | **49** | **50** | **51** | standard |
| --- | --- | --- | --- | --- | --- | --- | --- |
| **FUNGI** |  |  |  |  |  |  | *Amphotericin B* |
| *A. fumigatus* | **24.1±0.51** | **28.1±0.76** | **26.3±0.73** | 11.3±0.34 | **21.3±0.39** | 16.2±0.36 | 23.7±0.1 |
| *S. racemsum* | 16.9±0.52 | **23.4±0.77** | **20.9±0.61** | 12.1±0.25 | **17.2±0.16** | 15.0±0.44 | 19.7±0.2 |
| *G. candidum* | 11.5±0.43 | 14.1±0.65 | 12.6±0.54 | 15.3±0.38 | **24.6±0.58** | 17.6±0.58 | 28.7±0.2 |
| *C. albicans* | 10.8±0.46 | 13.2±0.58 | 11.2±0.44 | 12.6±0.34 | 11.5±0.3 | 13.7±0.5 | 25.4±0.1 |
| **(+) Gram Bacteria** |  |  |  |  |  |  | *Ampicillin* |
| *S. pneumoniae* | 16.3±0.42 | 14.6±0.58 | 18.2±0.68 | 15.0±0.43 | 18.2±0.4 | 16.9±0.58 | 23.8±0.2 |
| *B. subtilis* | 19.1±0.51 | 14.3±0.58 | 18.9±0.64 | 17.4±0.53 | 20.3±0.5 | 18.2±0.44 | 32.4±0.3 |
| **(-) Negative Bacteria** |  |  |  |  |  |  | *Gentamicin* |
| *P. aeruginosa* | 11.6±0.36 | 10.7±0.24 | **16.8±0.47** | 12.3±0.25 | 14.1±0.32 | **17.8±0.52** | 17.3±0.1 |
| *E. coli* | 10.9±0.21 | 9.9±0.34 | 13.9±0.54 | **17.8±0.32** | 15.9±0.64 | **18.5±0.61** | 19.9±0.3 |

**Table S6:** Antimicrobial activity of polyamides **52**-**57** (discs Ø 6mm).

| microorganism | **52** | **53** | **54** | **55** | **56** | **57** | standard |
| --- | --- | --- | --- | --- | --- | --- | --- |
| **FUNGI** |  |  |  |  |  |  | *Amphotericin B* |
| *A. fumigatus* | 16.5±0.58 | 12.6±0.38 | 11.5±0.58 | 12.7±0.38 | 15.7±0.47 | 10.9±0.32 | 23.7±0.1 |
| *S. racemsum* | 12.8±0.27 | 16.5±0.25 | **17.6±0.27** | 14.7±0.35 | 16.6±0.62 | 14.3±0.35 | 19.7±0.2 |
| *G. candidum* | 18.3±0.56 | **25.8±0.58** | **26.9±0.35** | 18.5±0.44 | 12.6±0.38 | 20.3±0.44 | 28.7±0.2 |
| *C. albicans* | 17.6±0.54 | 10.9±0.4 | 16.5±0.5 | 16.5±0.25 | 12.7±0.37 | 16.5±0.25 | 25.4±0.1 |
| **(+) Gram Bacteria** |  |  |  |  |  |  | *Ampicillin* |
| *S. pneumoniae* | **24.9±0.63** | 19.5±0.44 | **22.6±0.34** | 13.8±0.58 | 12.3±0.58 | **22.3±0.47** | 23.8±0.2 |
| *B. subtilis* | 21.5±0.34 | **29.8±0.58** | **33.7±0.25** | 11.7±0.58 | 12.7±0.37 | 19.7±0.43 | 32.4±0.3 |
| **(-) Negative Bacteria** |  |  |  |  |  |  | *Gentamicin* |
| *P. aeruginosa* | **22.7±0.56** | 12.3±0.25 | 13.1±0.32 | 10.9±0.4 | 9.8±0.34 | **18.8±0.36** | 17.3±0.1 |
| *E. coli* | **18.4±0.41** | 13.8±0.19 | 15.3±0.48 | 11.2±0.5 | 11.3±0.39 | **17.9±0.47** | 19.9±0.3 |

**Table S7** Statistical analysis of some polyamides exhibited promising antimicrobial agents

| **Polym.**  **No.** | **Affected**  **organism** | **Inhibition zone diameter**  **(mm)** | | **Statistical**  **analysis** | | **Action** |
| --- | --- | --- | --- | --- | --- | --- |
|  |  | **Compound** | **Reference** | **t** | **P** |  |
| **46** | *A. fumigatus* | 24.1±0.51 | 23.7±0.10 | 1.333 | 0.253 | Controls some fungi |
| **47** | *A. fumigatus* | 28.1±0.76 | 23.7±0.10 | 9.942^*^ | <0.001^*^ | Controls some fungi |
|  | *S. racemosum* | 23.4±0.77 | 19.7±0.20 | 8.056^*^ | <0.001^*^ |  |
| **48** | *A. fumigatus* | 26.3±0.73 | 23.7±0.10 | 6.112^*^ | 0.003^*^ | Controls some fungi |
|  | *S. racemosum* | 20.9±0.61 | 19.7±0.20 | 3.238^*^ | 0.031^*^ |  |
| **50** | *B. subtilis* | 33.7±0.25 | 32.4±0.30 | 5.766^*^ | 0.004^*^ | Gram positive antibiotic |
| **52** | *S. pneumoniae* | 24.9±0.63 | 23.8±0.20 | 2.882^*^ | 0.044^*^ | Broad spectrum antibiotic |
|  | *P. aeruginosa* | 22.7±0.56 | 17.3±0.10 | 16.442^*^ | <0.001^*^ |  |
| **53** | *P. aeruginosa* | 17.8±0.52 | 17.3±0.10 | 1.635 | 0.177 | Gram negative antibiotic |
| **57** | *P. aeruginosa* | 18.8±0.36 | 17.3±0.10 | 6.954^*^ | 0.002^*^ | Gram negative antibiotic |

t: Student t-test for comparing between compound and reference

*: Statistically significant at p ≤ 0.05
